# Laboratory Investigation of Simultaneous Ultraviolet Photoprocessing and Temperature-Programmed Desorption of Interstellar Ice Analogs

**DOI:** 10.1021/acsearthspacechem.5c00338

**Published:** 2026-01-30

**Authors:** Collette C. Sarver, Catherine E. Walker, Susanna L. Widicus Weaver

**Affiliations:** † Department of Chemistry, 5228 University of Wisconsin-Madison, Madison, Wisconsin 53706, United States; ‡ Departments of Chemistry and Astronomy, 5228 University of Wisconsin-Madison, Madison, Wisconsin 53706, United States

**Keywords:** Laboratory astrophysics, Interstellar medium, Dark interstellar clouds, Protoplanetary disks, Ice composition, Complex organic molecules

## Abstract

Ice mantles on dust grains in the interstellar medium and protoplanetary disks are sites that allow for complex chemistry to occur. The formation of interstellar complex organic molecules (iCOMs) in these astronomical environments relies on energetic processes such as photochemistry driven by ultraviolet (UV) photons and thermal processes. Simultaneous versus subsequent UV photoprocessing and temperature-programmed desorption (TPD) on pure methanol and methanol and water mixtures (14–21%) under laboratory conditions were studied to mimic conditions in dense clouds and disks. In experiments where the ice was irradiated and heated simultaneously, results suggest that comparing between different experiments with fluence but not flux or time held constant may be unreliable for mixed ices for some chemical species. This finding indicates that experiments might not be comparable to interstellar conditions if ice mixtures are involved. For experiments where the ice was irradiated and then warmed in sequential steps, the methanol and water ice mixtures show an enhancement of CO, H_2_CO, and CH_4_ production as compared to experiments with simultaneous irradiation and heating. Additionally, the production of more complex species (i.e., (CH_2_OH)_2_, HOCH_2_CHO, and CH_3_OCH_3_) is suppressed. This effect is best explained by the enhanced mobility of larger radicals with simultaneous heating and irradiation, yielding more complex molecules. Additional studies with a well characterized UV lamp are needed to explore this trend with other ice mixtures, photon fluxes, and fluences. Nonetheless, these results call into question a common assumption made in the study of ice analogs and may impact the interpretation of experimental and observational results.

## Graphical abstract

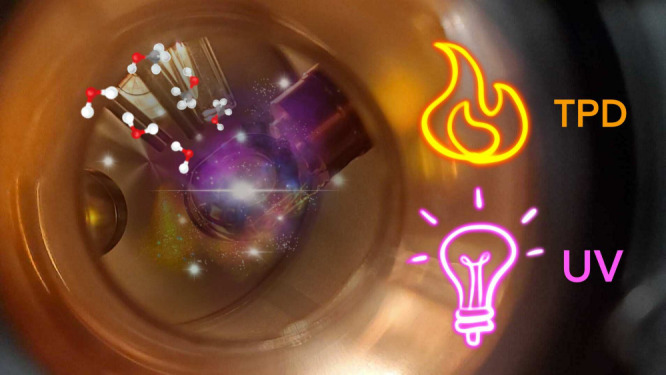

## Introduction

Interstellar complex organic molecules (iCOMs) are defined as molecules having six or more atoms and containing the element carbon.^1^ As of September 2025, more than 338 molecules have been detected in circumstellar environments or in the interstellar medium (ISM), with all molecules containing at least six atoms also being iCOMs.^1,2^ iCOMs are present both in the gas and solid phase in cold and dense neutral interstellar and circumstellar media. Although iCOMs can be formed in the gas phase, most complex chemistry occurs in the solid phase, where grain surface chemistry and ice sublimation dominate.^1^

iCOMs are observed in every stage of stellar evolution from molecular clouds to the formation of protoplanetary disks. The evolution of hot cores and corinos (prestellar cores differentiated by mass) can be separated into three phases where iCOM formation occurs: the cold phase, the warm-up phase, and the hot phase.^1^ During the cold phase, the cold cloud core ages and undergoes isothermal collapse at around 10 K.^3^ Grains of silicates and carbon serve as surfaces for atoms and molecules to stick and form icy mantles, predominantly made of water (H_2_O), carbon monoxide (CO), and carbon dioxide (CO_2_).^4–6^ Other species can accrete on the surface of these icy grains, including material formed from gas phase reactions, along with atomic hydrogen, oxygen, nitrogen, and carbon.^1^ Molecules undergo hydrogenation and oxygenation in the ice to form small volatiles such as water, methanol (CH_3_OH), carbon dioxide, ammonia (NH_3_), methane (CH_4_), and formaldehyde (H_2_CO).^1,7–9^ The warm-up phase occurs when material gravitationally collapses into a prestellar core and heats up from 10 K to 100–300 K.^10,11^ Radicals are produced through cosmic ray induced ultraviolet irradiation.^3^ As temperatures increase to 20 K or higher, these radicals diffuse on ice surfaces to form larger iCOMs like methyl formate (HCOOCH_3_), dimethyl ether (CH_3_OCH_3_), and acetaldehyde (CH_3_CHO), among others.^1,7,12^ Species undergo desorption via sublimation through thermal processing, or by nonthermal processes such as photodesorption and the ejection of molecules produced in exothermic surface reactions.^1,13^ Because of the relatively high temperatures of the hot core or corino, only gas-phase chemistry can occur during the hot phase, and species sublimate according to their surface binding energies.^11^ Unless the ice is incorporated into the cold midplane of the protoplanetary disk, ion–molecule and neutral–neutral reactions destroy or form additional iCOMs.^1,14^ Finally, the ices in the disk can undergo additional freeze-out or grain surface chemistry. The iCOMs which are formed in the cold midplane of the disk are of particular interest in the search for life.^1,15^

Icy planetesimals (comets) and planets form in the protoplanetary disk at temperatures around 20–40 K, within a few hundred AU of the protostar.^1^ iCOMs in this region are sometimes unaltered when they are incorporated into icy solar system bodies. Thus, this material is directly related to the chemical composition of the atmospheres of new planets.^1,16^ Many of the detected iCOMs, such as methanol, serve as parent species for biologically relevant molecules such as dimethyl ether and methyl formate, which may be essential in the origin of life.^16^ An unanswered question remains as to how these molecules survive to be delivered to planetary systems. Icy planetesimals share the same chemical composition as the late stages in planet formation and are thought to be the most pristine material preserved from the early solar system.^11,17^ Comets can then be accreted by planets to enrich their atmospheres with those iCOMs formed from icy dust grains. For example, the comet Shoemaker-Levy 9 collided with Jupiter in 1994, enriching the planet with previously undetected molecules like carbonyl sulfide (OCS) and carbon monosulfide (CS).^18^ Even with accretion efficiencies as low as 1%, exoplanets can still accrete cometary material on order of their own mass.^18^ By understanding the conditions of iCOM formation in early stages of star and planet formation, we can better discern the conditions required for the origin of life on our world and others.

To simulate interstellar ice chemistry, laboratory setups that can reach pressures below 1 × 10^–10^ Torr and temperatures as low as 10 K are needed. Different interstellar ice analogs can be deposited at these conditions depending on the type of system studied. The most abundant species in interstellar ices include H_2_O, CO, CO_2_, CH_3_OH, NH_4_, CH_4_, and cyanide-bearing molecules, where H_2_O is by far the most abundant ice component as seen by the Spitzer Space Telescope^5^ and the James Webb Space Telescope.^4^ In cold and dense regions, ice observations have determined that CH_3_OH abundance is typically between 1% to 30% of the total ice composition,^6^ with recent observations finding abundances closer to 10%.^4^

Numerous laboratory investigations have focused on the chemistry of ices under conditions simulating either processing of material in cold cores, or the warm-up of material to simulate formation of a hot core or processing of a comet. In such laboratory investigations, ice analogs are typically energetically processed by either irradiation by ultraviolet (UV) photons or bombarded with electrons or protons. Temperature-programmed desorption (TPD) heats the ice sample to simulate the warm-up that comets or icy grains experience when they move toward a protostar during cloud collapse and disk formation. The products formed from the energetic processing and TPD sublimate into the gas phase, where they can then further react. Previous studies have used infrared spectroscopy and mass spectrometry to analyze the solid ice during irradiation and its gas phase products during sublimation, respectively.^7,19–25^ The standard procedure for these experiments is to separate the physical processes and conduct UV irradiation and TPD (warm-up) in sequential steps.^8,22–27^ First, samples are held at cryogenic temperatures and irradiated. Then samples are warmed incrementally and both ice and gas are monitored. After all ice sublimates, the gas phase samples are analyzed. Although this is straightforward for sorting out the chemistry at each step of processing, it is not a realistic simulation of the processes that happen in environments in the interstellar medium (ISM). While there is indeed photoprocessing of ices when they are in cold clouds, during star-formation the warm-up occurs simultaneously with additional photoprocessing.

Herein we present the results of a series of experiments designed to examine the impact of simultaneous TPD and UV photoprocessing on simple interstellar ice analogs. We conducted these experiments to simulate conditions more closely resembling the real conditions in dense molecular clouds or protoplanetary disks. To do this, we used the Sublimation of Laboratory Ices Milimeter/submillimeter Experiment at the University of Wisconsin-Madison (SubLIME-UW), so the chemistry could be tracked in the ices via infrared spectroscopy and in the gas phase via mass spectrometry. UV photoprocessing and TPD experiments were conducted that explored the impact of photoprocessing of the ice before warm-up in two steps (henceforth referred to as “subsequent”) and during warm-up in one step (henceforth referred to as “simultaneous”). Since simultaneous UV photoprocessing and TPD may change the resultant chemistry by increasing the length of time for ice irradiation, i.e., the fluence, an additional set of experiments was conducted at a lower flux but similar fluence in the simultaneous trials.

## Experimental Methods

The SubLIME-UW instrument was used to study pure methanol and mixed ices containing methanol and water. The schematic for SubLIME-UW is shown in Figure 1.^22,23^ In these experiments, interstellar ice analogs were deposited and processed with UV irradiation and controlled warm-up within the chamber at temperatures and pressures as low as 10 K and 5 × 10^–11^ Torr. Liquid samples such as methanol (99.9% Sigma-Aldrich) and water (18.2 MΩ cm, Milli-Q Ultrapure Water System) were purified from atmospheric contaminants before deposition with two freeze–pump–thaw cycles using liquid nitrogen. To compare with previous SubLIME experiments, ices approximately 1000 monolayers thick of either pure methanol or homogeneous ice mixtures of 14–21% methanol in water were deposited. One monolayer is equivalent to a surface density of 1 × 10^15^ molecules/cm^2^. The formation of iCOMs from pure methanol has been studied before and provides a good foundation for comparison. Based on previous work,^25^ it has been seen that in a mixture of methanol and water, increasing the relative amount of water suppresses the formation of iCOMs and their spectral features become weak. Two sets of experiments with different concentrations of methanol were therefore performed to address the capture of small iCOM features with 100% methanol, and the more realistic interstellar ice of 14–21% methanol and water. The vapor samples were deposited through capillary tubes and high precision gas-dosing valves onto an IR-transparent KBr window substrate and polished gold sample substrate holder (Advanced ResearchSystems, Inc. model SHNO-1B). The cold arm of the substrate is a closed-cycle helium cryostat (APD Cryogenics 256844D1 expander model DE-202B) connected to a helium compressor (APD Cryogenics model HC-2). A temperature controller (LakeShore Model 335) with a silicon diode sensor (Scientific Instruments model SI-41) located at the tip of the cryostat arm varied the temperature of the substrate from cryogenic temperatures up to 310 K. A turbomolecular pump (Pfeiffer Vacuum, HiPace 700 backed by a turbo station: Pfeiffer Vacuum, HiCube 80 Eco) achieved the low pressures needed and was monitored constantly with a Bayard-Alpert ion gauge tube (Agilent model XGS-600). Ice samples were irradiated with a microwave-discharge hydrogen-flow lamp (MDHL). The MDHL was composed of a of a custom-blown quartz lamp in an F-type configuration, a microwave generator (Opthos Instrument Company, LLC, MPG-4 526), and a microwave McCarroll cavity. Microwaves generated inside the McCarroll cavity excite hydrogen gas flowing inside the lamp. A MgF_2_ window (MPF Products, INC.) on the lamp allows UV photons to enter the chamber while a quartz guidance tube directs the UV flux from the inside of the MgF_2_ window to the sample. After de-excitation, photons in the vacuum ultraviolet (VUV) range at wavelengths from 110 to 180 nm are emitted. The lamp’s spectrum exhibits strong Lyman-α (121.6 nm) emission and molecular hydrogen emission peaks from 140–170 nm. Previous work details the spectral distribution output as a function of wavelength of a typical MDHL in this configuration.^28^ This setup is not outfitted with a vacuum ultraviolet spectrometer to characterize this lamp specifically. Additionally, the setup contained a quadrupole mass spectrometer (QMS, Pfeiffer Vacuum, PrismaPro QMG 250 M2) that could probe mass-to-charge ratios up to 100 amu, and a Fourier-transform infrared spectrometer (FT-IR, Thermo Fisher Scientific, Nicolet iS50R FT-IR) with resolution of 0.241 cm^–1^. The beam of the FT-IR was enclosed with plastic and purged with dry nitrogen gas with additional desiccant present (Sigma-Aldrich, 4 Å molecular sieves) to reduce the effects of water and carbon dioxide interference from the atmosphere. Also present was a mm/submm light source paired with an InSb hot-electron bolometer detector to add rotational spectroscopy as a diagnostic tool, but this aspect of the experimental capabilities was not used in the work presented herein. Rotational spectroscopy is a valuable tool for molecular identification, but cannot currently be used simultaneously with mass spectrometry in the SubLIME-UW system due to pressure constraints.

**Figure 1:**
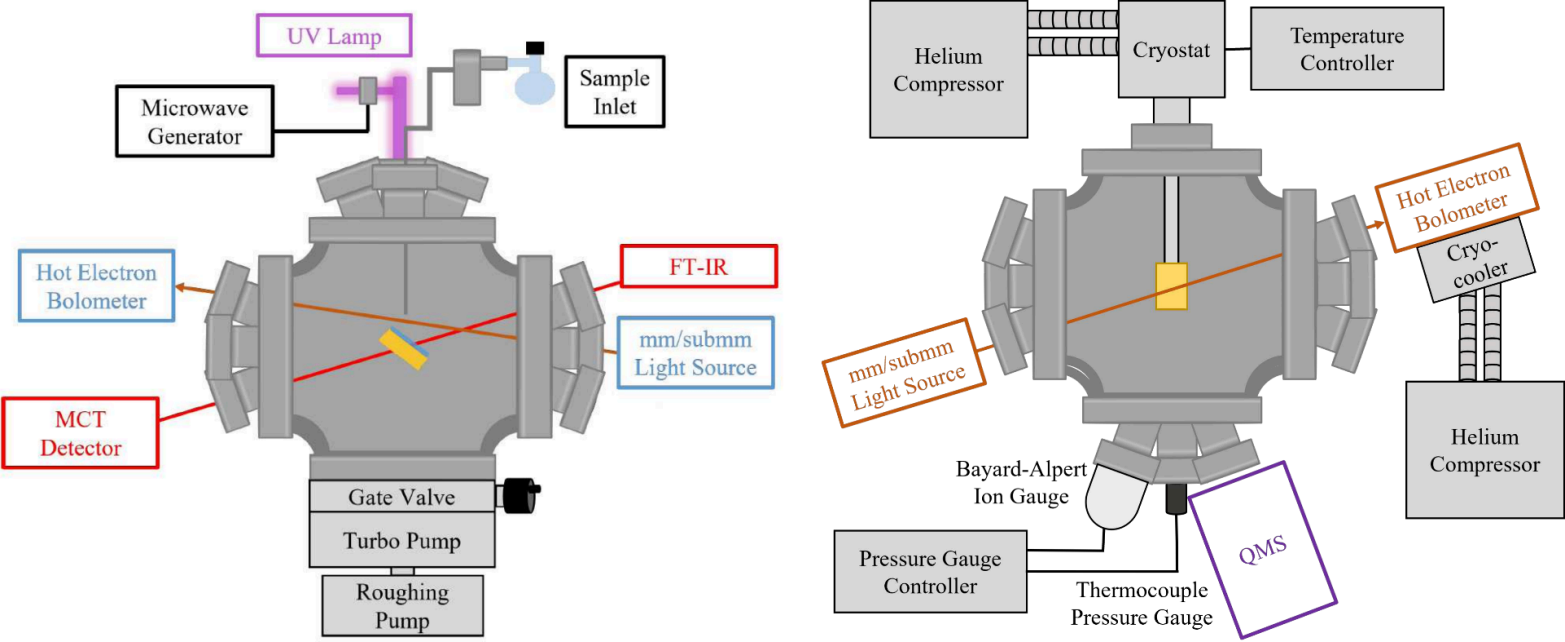
Side and top view of SubLIME.

The photon flux using the standard 100 W forward power setting from the microwave generator was measured to be 2.7(±0.7) × 10^14^ photons cm^–2^ s^–1^ using actinometry.^29,30^ Using the same method, a flux of 2.0(±0.3) × 10^14^ photons cm^–2^ s^–1^ was determined for a forward power setting of 50 W, which is a photon flux reduction of 26%. The photon flux measurement using this method is only accurate to an order of magnitude between different experimental setups, but it is a common assumption that such measurements give an accurate relative value when used within the same experimental setup. These two photon fluxes were selected so that it could be determined if the same photon fluence produces the same chemistry regardless of flux. At 100 and 50 W, the experiments had a total UV fluence of (4.5–4.9) × 10^18^ or (5.5–5.8) × 10^18^ photons cm^–2^ (hereafter referred to as “short” and “long” fluence, respectively), depending on whether there was an initial period of irradiation before TPD as has been done in previous experiments.^8,22–27^ As such, the “long” fluence experiments have simultaneous UV and TPD processing, but only after an initial period of UV photolysis at 10 K. These experimental fluences are comparable to conditions in a cold core after ∼15 million years, which is comparable to the typical lifetime of a prestellar core.^31^

To determine the effect of simultaneous UV and TPD processing on the production of species, a series of additional experiments were conducted at various equivalent fluence and temperature values using subsequent processing for both ice compositions. Depending on the corresponding fluence and temperatures, ices were irradiated with an equivalent fluence and then warmed up to the correct temperature in subsequent steps. For pure methanol ices, points at 20, 50, 100, 130, and 150 K were selected. For the methanol and water ice mixtures, points at 20, 50, 70, 100, 130, and 150 K were selected.

Table 1 lists experimental conditions and operating parameters when UV irradiation and TPD steps were completed simultaneously. For trials 1, 3, 5, and 7, a TPD ramp rate of 1 K/min was used and IR spectra were collected every 10 min, corresponding to a temperature increment of 10 K. These trials were conducted with a 100 W forward microwave generator setting, and a total UV photon flux of 2.7(±0.7) × 10^14^ photons cm^–2^ s^–1^. For trials 2, 4, 6, and 8, a TPD ramp rate of 0.8 K/min was used and IR spectra were collected every 12.5 min, corresponding to a temperature increment of 10 K. These trials were conducted with a 50 W forward microwave generator setting, and a total UV fluence of 2.0(±0.3) × 10^14^ photons cm^–2^ s^–1^. These flux and fluence values were selected to ensure the correct UV dosage per temperature unit. The QMS signal was collected throughout the entire experiment from deposition at 10 K to the end of warm-up at 310 K. Table 2 lists experimental conditions and operating parameters when UV irradiation and TPD were completed in sequential steps. In each trial, sample ices were irradiated with equivalent fluence corresponding to the desired temperature point in the simultaneous trials. Then samples were warmed up to the desired temperature and an IR spectrum was collected. Trials 1–4 and 9–13 were completed with pure methanol ices, and trials 5–8 and 14–19 were completed with 14–21% methanol mixed with water. Specific ice composition and thickness for each run included in the analysis can be found in the Supporting Information.

**Table 1:** A List of Experimental Trials and Important Operating Parameters for the Experiments with Simultaneous UV and TPD Processing

Trial	Methanol Percentage (%)	Time Photolyzed (min)	Microwave Generator Power (W)	UV Flux (photons cm^–2^ s^–1^)	UV Fluence (photons cm^–2^)
1	100	300	100	2.7 × 10^14^	4.9 × 10^18^
2	100	375	50	2.0 × 10^14^	4.5 × 10^18^
3	100	360	100	2.7 × 10^14^	5.8 × 10^18^
4	100	450	50	2.0 × 10^14^	5.5 × 10^18^
5	14–21	300	100	2.7 × 10^14^	4.9 × 10^18^
6	14–21	375	50	2.0 × 10^14^	4.5 × 10^18^
7	14–21	360	100	2.7 × 10^14^	5.8 × 10^18^
8	14–21	450	50	2.0 × 10^14^	5.5 × 10^18^

**Table 2:** A List of Experimental Trials and Important Operating Parameters for the Experiments with UV Processing Followed by Subsequent TPD Processing^a^

Trial	Methanol Percentage (%)	Time Photolyzed (min)	UV Fluence (photons cm^–2^)	Temperature (K)
9	100	24	1.5 × 10^17^	20
10	100	93	6.2 × 10^17^	50
11	100	206	1.4 × 10^18^	100
12	100	406	1.9 × 10^18^	130
13	100	447	2.2 × 10^18^	150
14	14–21	20	1.5 × 10^17^	20
15	14–21	99	6.2 × 10^17^	50
16	14–21	148	9.3 × 10^17^	70
17	14–21	247	1.4 × 10^18^	100
18	14–21	345	1.9 × 10^18^	130
19	14–21	377	2.2 × 10^18^	150

a All trials were completed with a forward microwave generator power of 100 W.

## Results & Discussion

### Infrared Spectra

The infrared spectra of the sample before and after irradiation with two different photon fluxes but before simultaneous TPD are shown in Figure 2, corresponding to the long fluence cases (trials 3, 4, 7, and 8; refer to the Experimental Methods section for their description). Both pure methanol ices and the 14–21% methanol ice mixtures are shown in Figure 2. This enables comparison of only the photon induced products before TPD start at equivalent fluence with two photon fluxes to determine if it yields the same products at the same rates. An increase in peak absorbance in the infrared spectra indicates the formation of products. Observed spectral features from products resulting from the irradiation of pure methanol were CO_2_ at 2341 cm^–1^;^32^ CO at 2135 cm^–1^;^32^ H_2_CO at 1722 cm^–1^ (with contributions from H_2_CO,^33^ CH_3_CHO;^34^ and HCOOCH_3_ ^35^), 1500 cm^–1^,^33^ and 1248 cm^–1^;^33^ CH_4_ at 1302 cm^–1^;^32,36^ and CH_3_OCH_3_ at 1162 cm^–1^ and 920 cm^–1^.^7,34^ The photon induced destruction of CH_3_OH is seen in the decreased absorbance of 1020 cm^–1^.^37^ These products have previously been observed in the irradiation of pure methanol ice.^7,22,24,25,33–35,37^ The methanol and water mixed ice yielded similar products. The exception was CH_3_OCH_3_ at 1162 cm^–1^ and 920 cm^–1^, which was not observed. It has been shown in previous studies of methanol and water ice mixtures that the addition of water to methanol ices suppresses the formation of iCOMs.^25,38^ The products produced in both ice compositions with high and low flux look similar, with the same band positions and the same overall absorbance. However, these IR spectra are not normalized with respect to the initial amount of methanol present, so additional comparisons are made with normalized production plots below.

**Figure 2:**
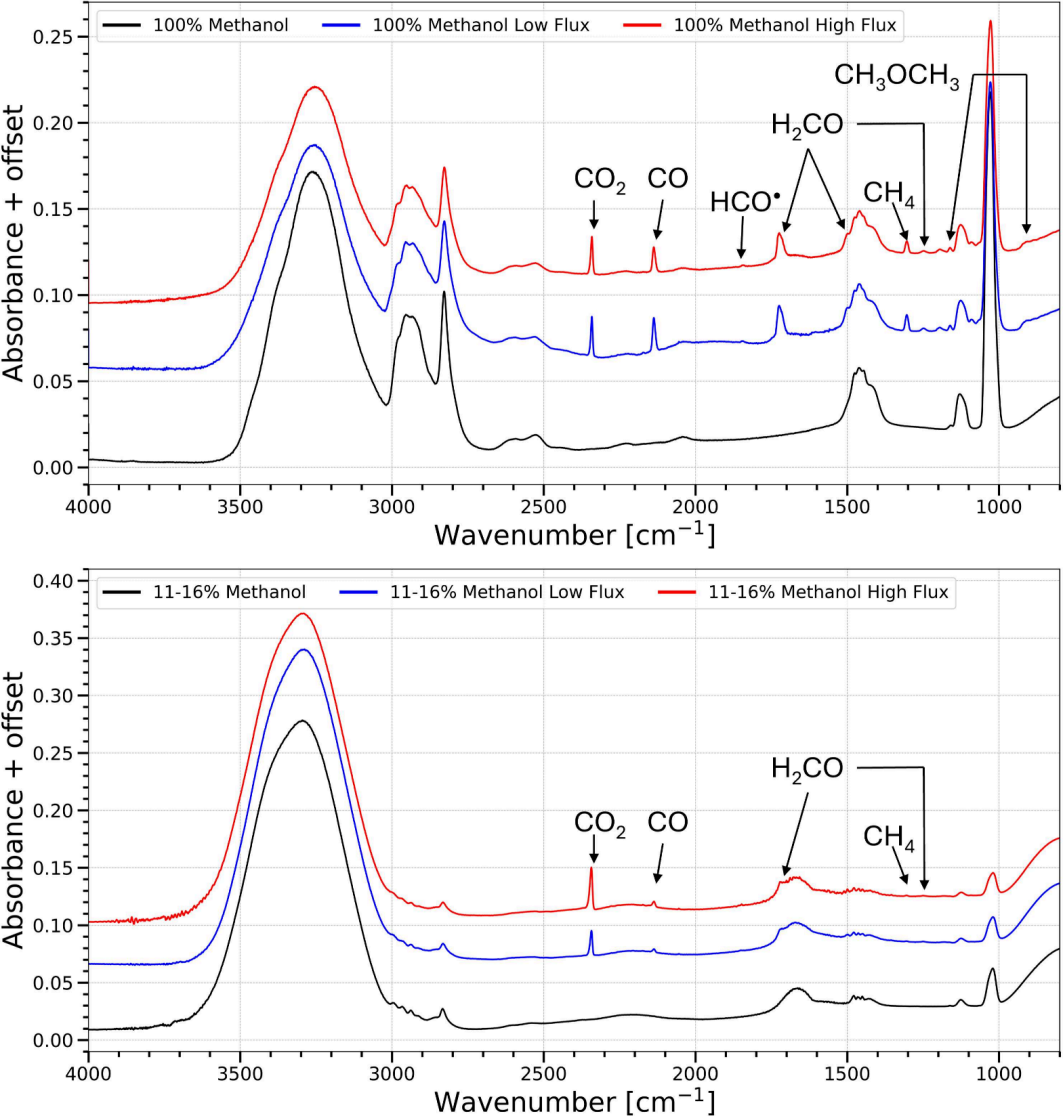
Infrared spectra of a pure methanol ice (top, trials 3 and 4) and 15 ± 4% and 14 ± 3% methanol in water mixed ice (bottom, trials 7 and 8) before (black) and after (blue and red) a UV fluence ranging from 9.1 to 9.7 × 10^17^ photons cm^–2^ but before simultaneous TPD at 10 K. Major products have been labeled. Spectra have been offset for clarity.

Figure 3 shows the infrared spectrum from one trial of pure methanol ice (left, trial 1) and 16(±4)% methanol ice (right, trial 5) every 10 K for the duration of simultaneous irradiation and warm-up. The first spectrum at 10 K in dark blue is the sample ice deposited before irradiation and warm-up. The final red spectrum is the resulting IR spectrum at 310 K after total irradiation and warm-up has occurred simultaneously. The IR spectra for each simultaneous trial type is listed in the Supporting Information.

**Figure 3:**
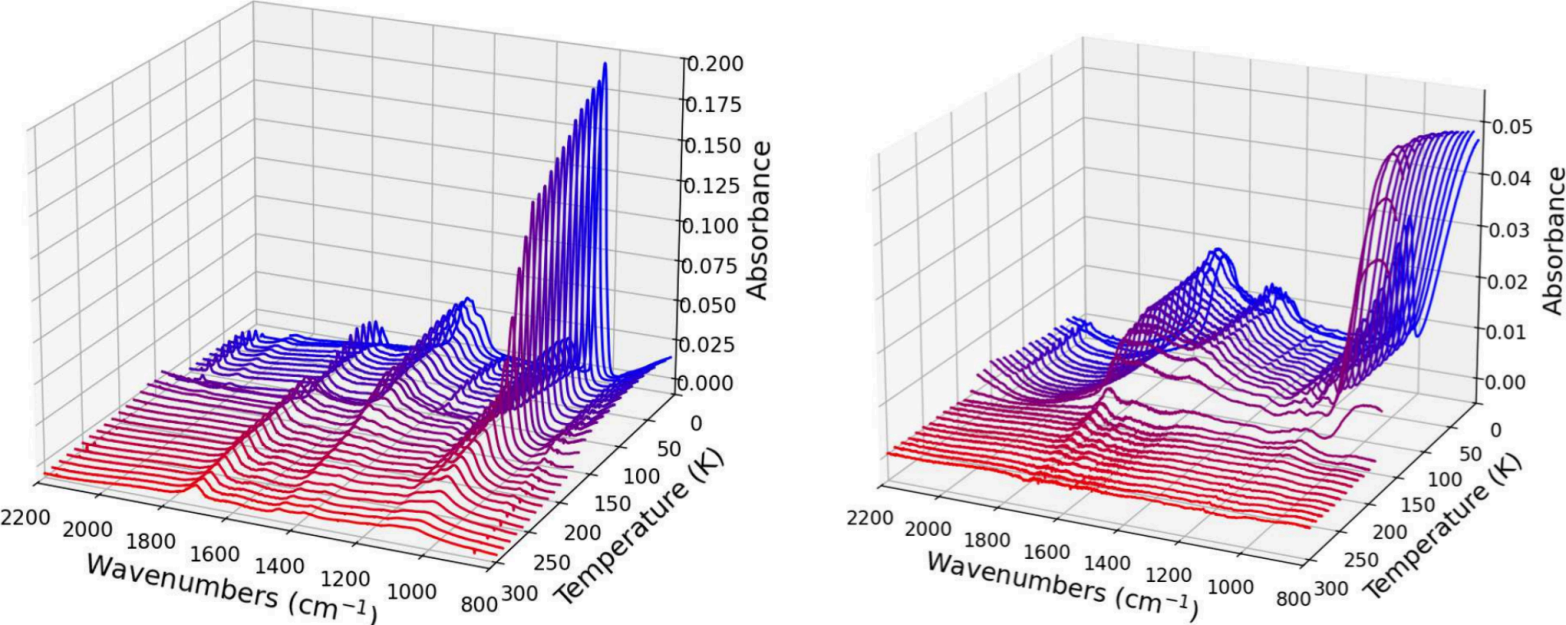
Infrared spectra of a pure methanol ice (left) and 16 ± 4% methanol in water mixed ice (right) from 10 to 310 K under simultaneous UV photoprocessing and TPD. The wavelength range is from 800 to 2200 cm^–1^ for clarity.

After simultaneous irradiation and heating, a residue was present on the substrate at 310 K in the experiments using pure methanol ices; the IR spectrum of this residue is seen in Figure 4. This residue was not observed in experiments using realistic interstellar ice mixtures of methanol and water. This could simply be due to the fact there is less methanol in these ice mixtures, and thus the residue is below the limit of detection. Trials 1–4 show very similar residual spectra regardless of fluence or photon flux. Table 3 lists the spectral features in Figure 4 and potential vibrational mode assignments for the residual material.

**Figure 4:**
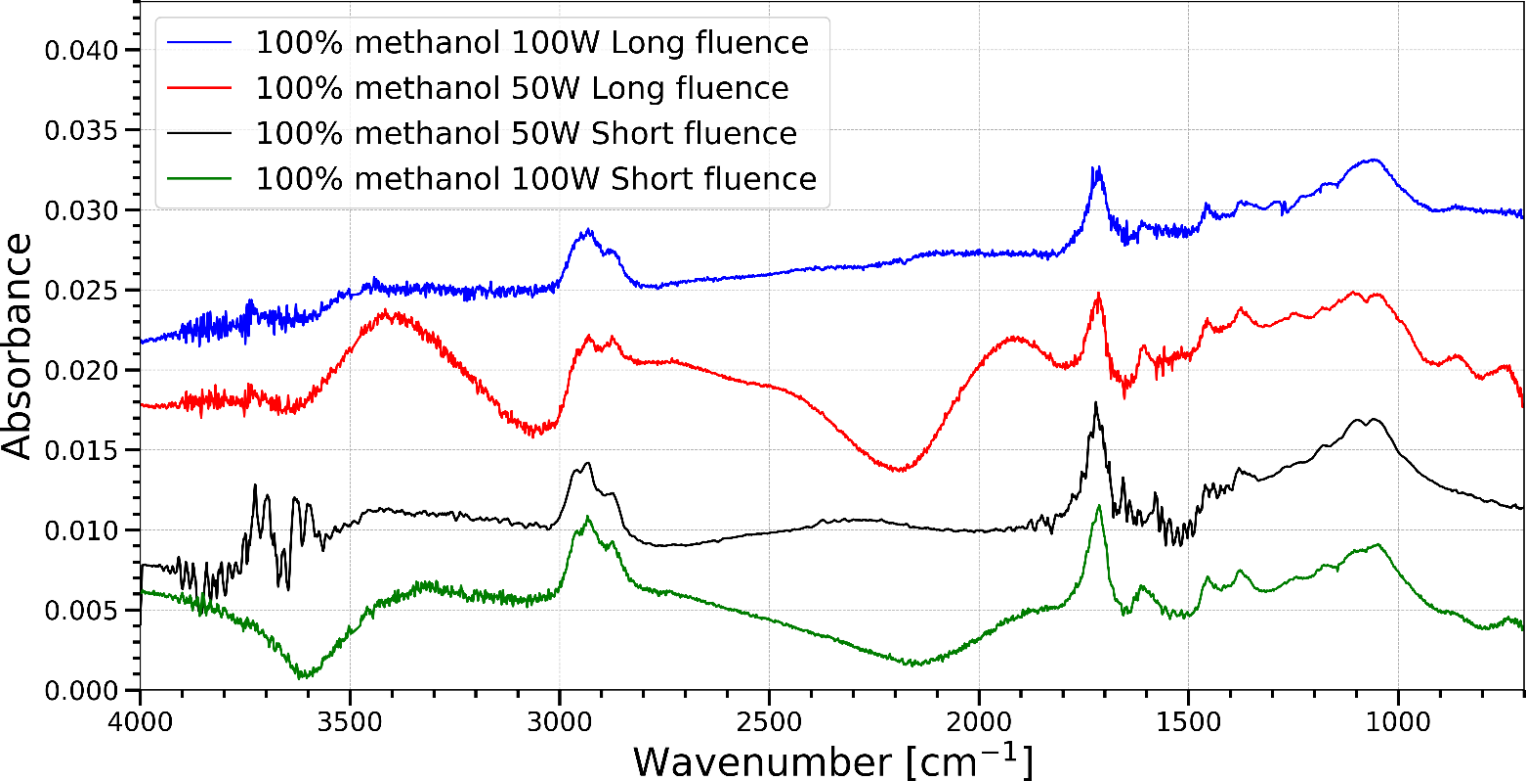
Infrared spectra of residue remaining from pure methanol ices after simultaneous TPD and photoprocessing. Spectra were collected when the sample was at 310 K. The rolling baseline is due to a change in atmospheric water between the background and 310 K. Short fluence refers to a total UV fluence of (4.5–4.9) × 10^18^ photons cm^–2^, while long fluence refers to a total UV fluence of (5.5–5.8) × 10^18^ photons cm^–2^. Spectra have been offset for clarity.

**Table 3:** Absorption Bands Present in the Residue That Remained at 310 K after Simultaneous UV and TPD of Pure Methanol Ices^39^

Peak Position (cm^–1^)	Vibrational Modes
2932	C—H stretching
2875	C—H stretching
1713	C=O stretching
1607	C=C stretching
1455	C—H bending
1376	C—H bending
1245	C—O stretching
1178	C—O stretching
1095	C—O stretching
1049	CO—C—CO stretching or C—O stretching
857	C—H bending
743	C—H bending

### Normalized Integrated Absorbance Plots

Figure 2 shows the IR spectrum after irradiation of both ice compositions using similar fluences but before simultaneous TPD at 10 K. In both cases, the blue and red spectra are nearly identical, supporting the assumption that experiments conducted using the same fluences, set to a value similar to astronomical conditions, should produce the same chemistry.^7^ However, a closer look at the change in normalized absorbance in all trials taken as a function of UV fluence challenges this assumption. Figures 5–14 track the change in integrated absorbance of a particular infrared feature as a function of UV fluence and associated temperature. All integrated absorbance measurements (*N*_*t*_) were normalized to the initial amount of methanol in each sample by the integrated absorbance of the C–O stretching vibrational mode of CH_3_OH at 1020 cm^–1^ at time *t* = 0 after deposition (*N*_0_). This yields *N*_*t*_/*N*_0_ on the *y*-axis. The normalized integrated absorbance as a function of UV fluence traces the chemistry (rate of destruction, production, or sublimation of a species) over the course of the experiment. Presented here are plots of particular absorption bands present in both the pure methanol and methanol and water mixed ices for both short fluences (trials 1, 2, 5, and 6) and long fluences (trials 3, 4, 7 and 8), as well as the equivalent subsequent UV and TPD runs (all trials in Table 2). All error bars associated with the normalized integrated absorbance are based on the random instrumentation error of the FT-IR. To determine this error, a sample ice with infrared features in each frequency area of interest was deposited and 30 infrared spectra were taken in sequence without changing the conditions of the sample. The standard deviation of spectral intensity for that set was calculated and then the corresponding uncertainty for any single integrated absorbance measurement was determined. The error was then propagated for each measurement by normalizing to the initial amount of methanol. The random instrumentation error changes as a function of frequency (primarily due to atmospheric water absorption), so each band of interest had a corresponding error applied based on the wavenumber range. This error was used in all subsequent plots since it was determined to be greater than the error associated with the noise of the baseline. Additionally, each point has an associated fluence uncertainty in the *x*-axis due to the uncertainty in the flux calculation. An uncertainty of 25% and 15% was assigned to the high and low flux measurements, respectively. These flux uncertainties were not added to the plots to aid in legibility. Traces that are labeled only with the microwave generator forward power setting of 50 or 100 W are trials in which UV and TPD occurred simultaneously (trials in Table 1). The traces that are labeled “subsequent” indicate that the UV and TPD were completed in subsequent steps (trials in Table 2). Plots labeled “long fluence” correspond to trials where there was an additional period of irradiation before simultaneous UV and TPD.

**Figure 5:**
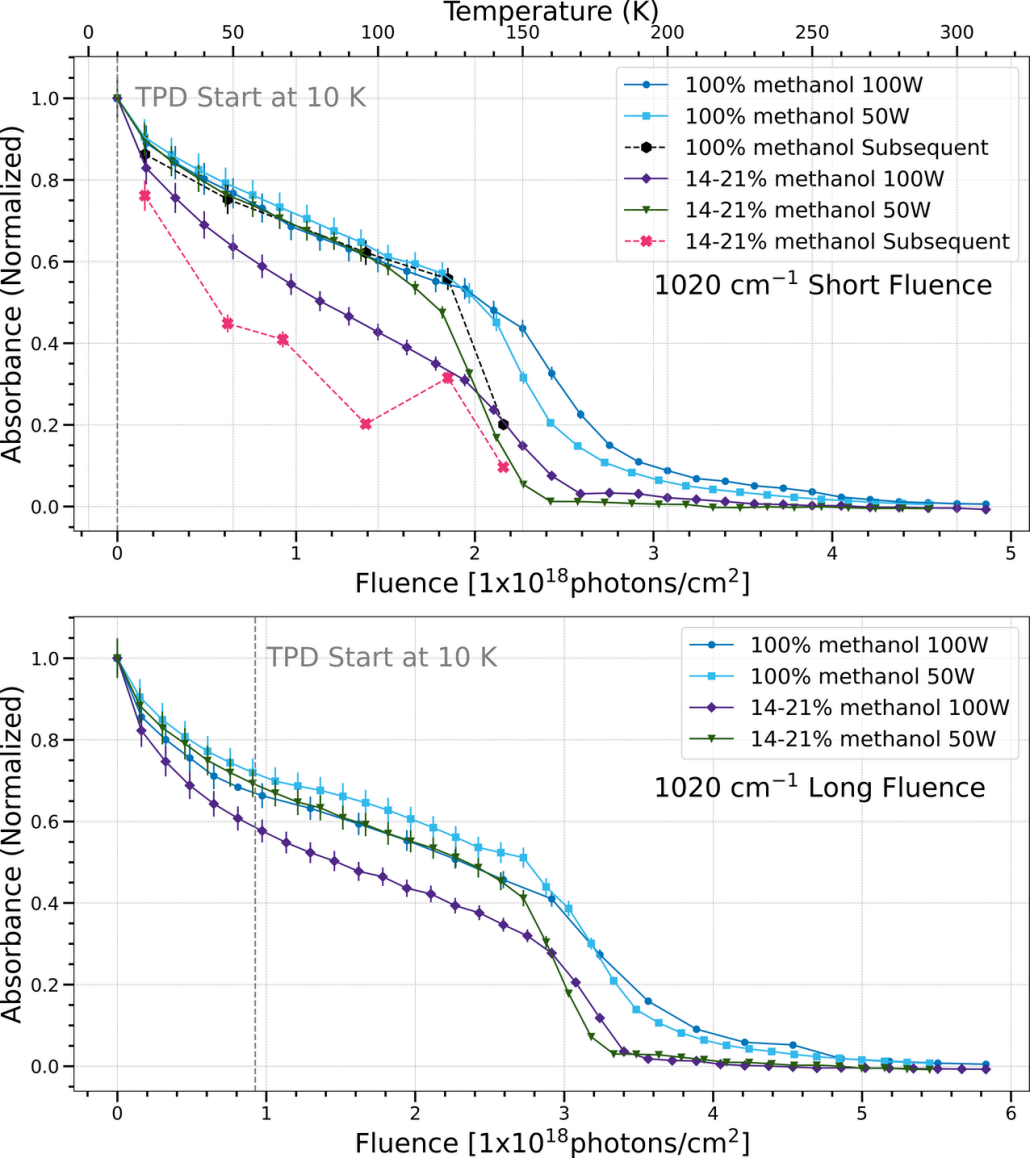
Change in normalized integrated absorbance of the 1020 cm^–1^ band as a function of UV fluence and temperature. Short fluence refers to a total UV fluence of (4.5–4.9) × 10^18^ photons cm^–2^ (top), while long fluence refers to a total UV fluence of (5.5–5.8) × 10^18^ photons cm^–2^ (bottom).

**6:**
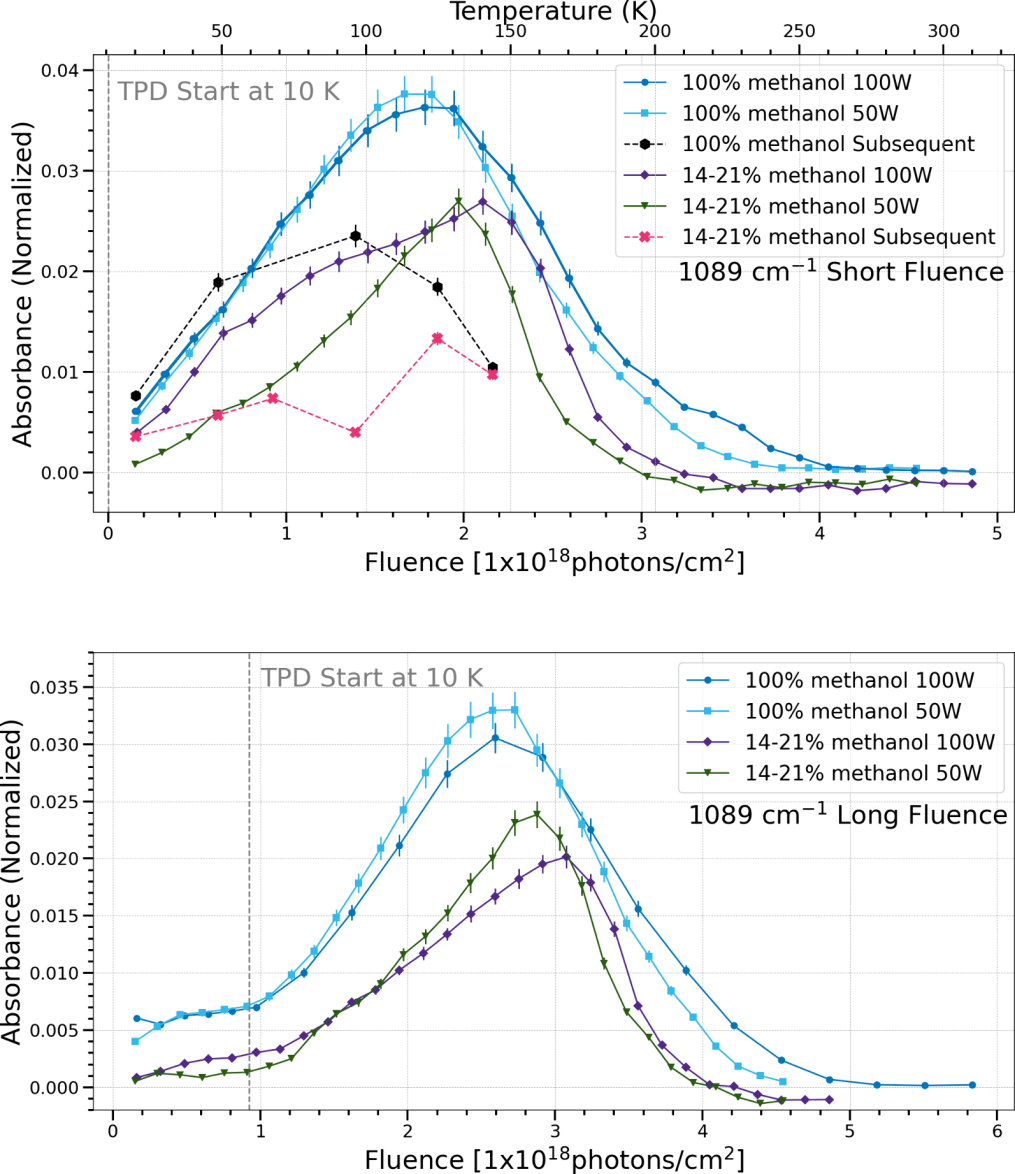
Change in normalized integrated absorbance of the 1089 cm^–1^ band as a function of UV fluence and temperature. Short fluence refers to a total UV fluence of (4.5–4.9) × 10^18^ photons cm^–2^ (top), while long fluence refers to a total UV fluence of (5.5–5.8) × 10^18^ photons cm^–2^ (bottom).

**7:**
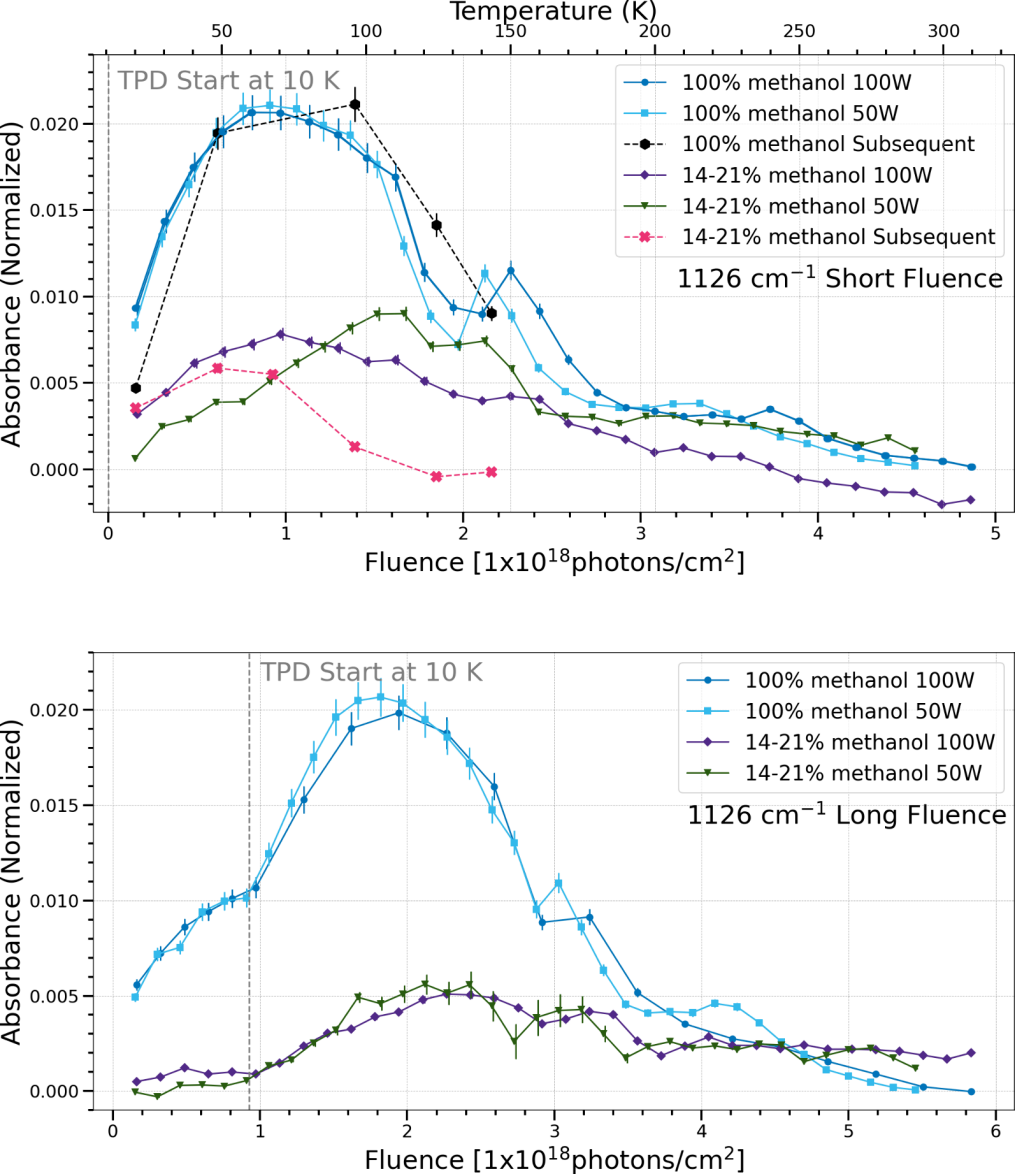
Change in normalized integrated absorbance of the 1126 cm^–1^ band as a function of UV fluence and temperature. Short fluence refers to a total UV fluence of (4.5–4.9) × 10^18^ photons cm^–2^ (top), while long fluence refers to a total UV fluence of (5.5–5.8) × 10^18^ photons cm^–2^ (bottom).

**8:**
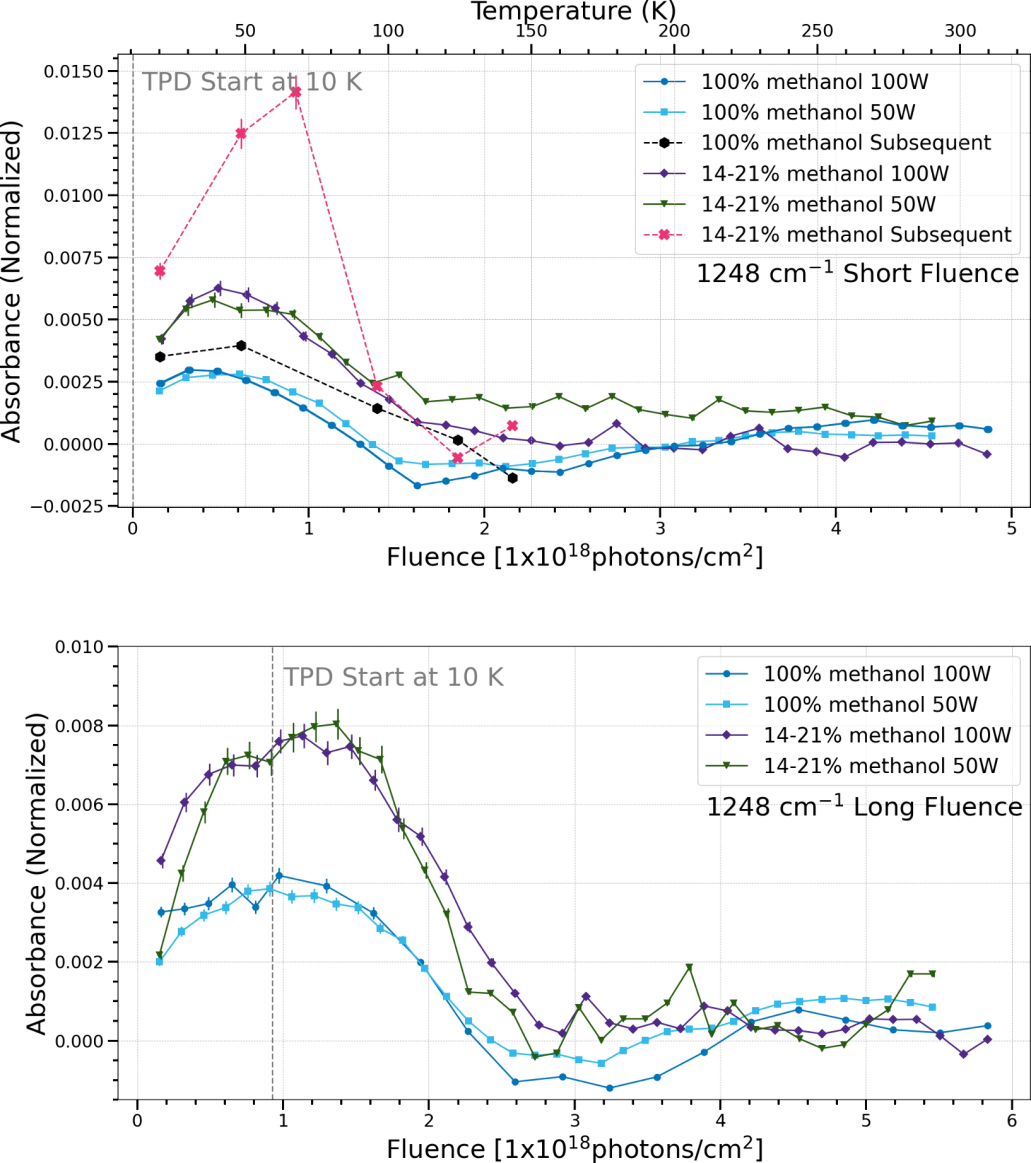
Change in normalized integrated absorbance of the 1248 cm^–1^ band as a function of UV fluence and temperature. Short fluence refers to a total UV fluence of (4.5–4.9) × 10^18^ photons cm^–2^ (top), while long fluence refers to a total UV fluence of (5.5–5.8) × 10^18^ photons cm^–2^ (bottom).

The 1020 cm^–1^ band is attributed to the C–O stretching vibrational mode in methanol. The pure methanol ices show a similar trend of destruction and sublimation as the fluence increases, regardless of photon flux (i.e., both blue traces). This is consistent with previous findings.^7^ The destruction and sublimation rate of the methanol and water mixed ices show differing trends, with a higher UV flux (i.e., purple traces) destroying methanol faster than a lower flux (i.e., green traces). This trend holds for both the short and long fluence periods investigated. The black trace in the short fluence case corresponds to pure methanol ices with subsequent photoprocessing and warm-up. The rate of methanol destruction and sublimation follows well with the simultaneous traces up until the bulk ice sublimation around 150 K, in which case it sublimates faster. The pink traces represent 14–21% methanol and water mixtures with subsequent UV and TPD steps. These traces show the most differences from the simultaneous trials, with all temperature points showing a more dramatic methanol destruction and sublimation rate.

The 1089 cm^–1^ band is attributed to many complex organic molecules such as dimethyl ether, glycolaldehyde, and ethylene glycol, instead of a single species.^22,34^ As with the 1020 cm^–1^ band, the pure methanol ices tend to show the same production and sublimation regardless of photon flux, while the 14–21% methanol ices show seemingly different behavior depending on flux. Pure methanol ices produce more complex molecular products than do the mixed ices, consistent with previous findings.^25^ However, there is a large difference in production and sublimation of this band between subsequent and simultaneous trials. During subsequent trials (black and pink traces), the peak production of the 1089 cm^–1^ band before sublimation occurs at lower temperature at approximately 80 K. In the simultaneous trials, the peak production of this band before bulk ice sublimation occurs at approximately 120 K. This indicates the simultaneous trials enhance entrapment and production of iCOMs.

Since the 1126 cm^–1^ band overlaps with a vibrational mode of methanol, the initial deposition spectrum of the ice was subtracted from subsequent spectra based on the percentage of remaining methanol at each temperature using the 1020 cm^–1^ band. If this band was attributed solely to methanol, there should be a decrease in normalized integrated absorbance as the methanol is photodissociated (see Figure 5). However, caution must be used when drawing conclusions solely on the use of infrared bands that overlap with the vibrational modes of methanol, including bands 1126, 1428, and 1460 cm^–1^ presented in this work. There may be additional contributions from other products and potentially the recombination of methanol, causing an underestimation of methanol destruction and an overestimation of product growth in these regions.

The 1126 cm^–1^ band is attributed to either the CH_3_ rock + C—C stretching + C—C=O bending of acetaldehyde^34^ or a vibrational mode of methoxymethanol.^25,38^ In the short fluence case, the simultaneous pure methanol ices show a similar trend regardless of flux, while the methanol and water mixed ices show a differing trend based on low or high flux. The pure methanol ices show the same trend regardless of simultaneous or subsequent processing. The methanol and water ice mixtures sublimate at lower temperatures in the subsequent processing case than for the simultaneous processing case. In the long fluence case, both ice compositions show similar trends regardless of high or low photon flux.

The band centered at 1248 cm^–1^ is attributed to the CH_2_ rock of formaldehyde and the CH_3_ rock of dimethyl ether.^33,34^ Both the pure methanol ices and the methanol and water mixed ices show a similar trend in production and sublimation regardless of flux for the simultaneous trials. This is valid for both fluence lengths investigated. In the subsequent cases, there is enhanced production of 1248 cm^–1^ for both ice compositions. This band is particularly enhanced in the methanol and water ice mixtures, similar to the CO band at 2135 cm^–1^ in Figure 14.

The band centered at 1302 cm^–1^ is attributed to the antisymmetric vibrational bending mode of methane.^36^ The pure methanol ices show a similar trend in formation and sublimation as fluence increases regardless of flux for both long and short fluence in the simultaneous processing trials. The methanol and water ices show more methane production with higher flux for both fluence cases. In subsequent processing trials, there is enhanced methane production as compared to the simultaneous processing trials for both ice compositions.

The band centered at 1428 cm^–1^ overlaps with a vibrational mode of methanol and thus subtracted spectra were used to determine the change in integrated absorbance of this band. It may be the CH_3_ deformation of acetaldehyde.^34^ There may be additional band carriers that are possibilities, but identification is unclear. Nonetheless, in the short fluence case for simultaneous processing, both pure methanol ice and methanol and water ice mixtures show similar trends regardless of photon flux. In the long fluence case, the pure methanol ices show similar production and sublimation while the methanol and water ice mixtures show a slightly different rate depending on photon flux, with a lower photon flux producing more of this band. In the subsequent processing trials, the production and sublimation of this band follow similar trends to their simultaneous processing counterparts for both ice compositions. The exception is at 150 K for each, when the subsequent processing trials sublimate faster than the simultaneous processing.

The 1460 cm^–1^ band can be attributed to the vibrational modes of dimethyl ether^34^ and ethylene glycol.^25^ Both ice compositions show similar trends regardless of photon flux in the simultaneous processing trials for both the long and short fluence cases. The subsequent processing trials have weak or no signals at 1460 cm^–1^. In every other case, if the realistic ice mixtures produced more of a particular species than the pure methanol ices in the simultaneous trials, then it would exhibit the same trend in the subsequent trials. This is the exception, where the methanol and water ices produced a stronger signal at 1460 cm^–1^ in the simultaneous processing trials as compared to those for pure methanol, but less in the subsequent processing trials. Additionally, the production of the 1428 and 1460 cm^–1^ bands seems to be thermally dependent, with production rates increasing in the long fluence case only after TPD begins. Again, caution should be used with these bands since both overlap with a vibrational mode of methanol.

The band centered at 1500 cm^–1^ is attributed to the CH_2_ scissoring in formaldehyde.^33^ Both the pure methanol and mixed methanol and water ices show similar trends in production and sublimation regardless of photon flux in both fluence cases, and for both simultaneous and subsequent processing trials.

The broad band centered at 1722 cm^–1^ is attributed to the C = O stretching in many different species including formaldehyde, methyl formate, and acetaldehyde.^33–35^ Processing of pure methanol ices produced an additional peak in this region at 1746 cm^–1^, which is attributed to glycolaldehyde.^40^ It was fitted separately and subtracted from the 1722 cm^–1^ band in trials using pure methanol ices. The production and sublimation of this band can be seen in the Supporting Information. In the short and long fluence cases, the pure methanol ices show a similar production and sublimation regardless of photon flux. The 14–21% methanol and water ices seem to have differing trends depending on photon flux, with more production arising from higher flux, particularly in the long fluence case. In subsequent processing trials, the production and sublimation of the 1722 cm^–1^ band was generally the same for both ice compositions, except the pure methanol ices sublimated at a lower temperature.

The 2135 cm^–1^ band is attributed to the only vibrational mode in CO, C=O stretching.^32^ In the simultaneous trials, the pure methanol ices show the same trend of production and sublimation regardless of flux in both fluence cases investigated. The mixed methanol and water ices show differing trends at both short and long fluence, with a higher photon flux forming more CO before sublimation. Overall, a lower percentage of methanol with a high flux produced the most CO out of all of the simultaneous processing trials. In the subsequent processing trials, the pure methanol ice produced and sublimated CO at the same rate as the simultaneous processing trials. The methanol and water mixtures produced more than the pure methanol ices in all cases. In the subsequent processing trials, a substantial enhancement of CO production occurred in the trials using water and methanol mixtures.

Considering the short fluence case, five bands (1020 cm^–1^, 1089 cm^–1^, 1302 cm^–1^, 1126 cm^–1^, 2135 cm^–1^) show that the chemistry in pure methanol ices under different photon fluxes is the same, while the methanol and water ice mixtures show a difference in chemistry with different photon fluxes in the simultaneous procssing trials. Meanwhile, four bands (1500 cm^–1^, 1248 cm^–1^, 1722 cm^–1^, 1428 cm^–1^, 1460 cm^–1^) show the same chemistry for both types of ices regardless of flux.

To further investigate these trends, additional comparisons were made for bands that were only present in the pure methanol ices. Six additional bands were integrated, of which four have blended transitions. These plots can be found in the Supporting Information. In pure methanol ices, five out of the six bands show the same production and sublimation regardless of photon flux for both the short and long fluence cases: 910/920 cm^–1^ (O–CH_3_ stretch of HCOOCH_3_ ^22,35^ and CH_3_OCH_3_ ^34^), 1162 cm^–1^ (CH_3_ rock of dimethyl ether^34^), 1195/1212 cm^–1^ (blended band attributed to the C–O stretching of the CH_2_OH radical^22^ and potentially HCOOCH_3_ ^7,25^ ), 1340/1376 cm^–1^ (blended band of potentially CH_3_CHO, C_2_H_6_, and CH_3_CH_2_OH^7^ or (CH_2_OH)_2_ and HCOCH_2_OH)^40^), and 1746 cm^–1^. Only one shows the same at short fluences and differ at long fluence: 886 cm^–1^ ((CH_2_OH)_2_ and HCOCH_2_OH)^40^). From these fluence vs integrated absorbance plots, it generally seems that pure methanol ices tend to produce the same chemistry under similar fluences in agreement with previous work.^7^ However, this work illustrates the trend of mixtures of water and methanol ices showing different chemistry for some species under the same fluence conditions with simultaneous UV irradiation and TPD.

The subsequent processing trials give results that differ significantly from their equivalent simultaneous processing trial counterparts. IR bands that are enhanced in the pure methanol ices during subsequent trials are 910/920 cm^–1^, 1248 cm^–1^ (formaldehyde), 1302 cm^–1^ (methane), and the enhanced destruction of 1020 cm^–1^ (methanol). In the mixed methanol and water ices, there is enhanced production of the 1248 cm^–1^ (formaldehyde), 1302 cm^–1^ (methane), and 2135 cm^–1^ (CO) bands, and the enhanced destruction of methanol. We turn to formation mechanisms to explain these trends and consider the reaction network presented in previous work of observed products of pure methanol after UV irradiation.^7^ The simpler species such as H_2_CO, CO, and CH_4_ all result from the photolysis of methanol. This leads to hydrogen abstraction, and does not require larger radicals than H atoms to form these products. Out of all the pathways available to methanol from UV irradiation, the ones that do not require the mobility of larger radicals are favored, resulting in an enhancement of those simpler species.^7^

On the other hand, the subsequent trials also showed a suppression of the production of the 886 cm^–1^ ((CH_2_OH)_2_, HOCH_2_CHO), 1089 cm^–1^ (HOCH_2_CHO, CH_3_OCH_3_), and 1460 cm^–1^ (CH_3_OCH_3_, (CH_2_OH)_2_) bands for the pure methanol ices. For the water and methanol ice mixtures, there was a suppression of the production of the 1089 cm^–1^, 1126 cm^–1^ (CH_3_CHO, CH_3_OCH_2_OH), and 1460 cm^–1^ bands. These bands of larger organics require larger radicals than hydrogen to form. For example, the last step in the formation pathway for HCOCH_2_CHO requires the addition of CHO, (CH_2_OH)_2_ requires CH_2_OH, and CH_3_OCH_3_ requires CH_3_O.^7^ Overall, the subsequent processing trials enhance the production of simple species and suppress the production of more complex species. The most likely explanation for this observation involves the mobility of the radicals and molecules in the binding sites on the surface of the ices.^13,41^ In the subsequent trials, the radicals made from the UV photolysis are essentially trapped in the binding sites at 10 K, with the exception of hydrogen. When heating is applied in the subsequent trials after photolysis, the smaller radicals make simpler species. In the simultaneous trials, the added heating allows for larger radical mobility, yielding an enhanced production of iCOMs.^13,41^

Furthermore, there are differences between the simultaneous trials of pure methanol and the ice mixtures with different photon fluxes but equivalent fluences. The methanol destruction rate was enhanced in the water mixtures as compared to the pure methanol ices due to the increased presence of H and OH radicals from H_2_O photolysis (Figure 5). The likelihood of methanol recombining after dissociation is lower when surrounded by H and OH radicals. Additionally, kinetics comes into play when considering the H abstraction reactions involving the radical products of methanol photodissociation such as CH_2_OH and CH_3_O. Due to the size of the electron orbitals, H abstraction from the carbon is more likely than from the oxygen since the absorption cross section of CH_3_OH is higher in the spectral region of C–H bond breakage than the O–H region.^42,43^ Statistically, there are also more C–H bonds to break than O–H bonds. This H abstraction would also be enhanced when there are more H and OH radicals present, like in the case of the methanol and water ice mixtures. Then the difference in destruction rate between the higher and lower flux cases results directly from a higher flux being able to photodissociate methanol more efficiently than the lower flux, regardless of equivalent fluence. In the case of CH_4_, the pure methanol ices led to a higher production than the water and methanol mixtures (Figure 9). There is one pathway for formation, starting with methanol, that yields CH_3_ and adds a H to form CH_4_.^7^ The water mixture forms more CH_4_ due to more H being present in the ice from water photolysis. Then higher flux produces more of those radicals than lower flux, regardless of equivalent fluence, yielding more CH_4_ in the 100 W trial than the 50 W trial. For H_2_CO formation, the mixed ices produced more than the pure methanol ices. This is due to an additional formation pathway available with the addition of water with CH_2_OH + H_2_O_2_ yielding H_2_CO + OH.^38^ Finally, there was more production of CO in the mixed ices than the pure methanol ices (Figure 14). Revisiting the reaction network of observed products of pure methanol after UV irradiation,^7^ there are two reaction pathways available for HCO. One results in the formation of HOCH_2_CHO, and the other results in the formation of CO. If there is less methanol present, the pathway that results in HOCH_2_CHO is less favored since it requires CH_2_OH, leading instead to enhanced CO production. Higher flux again favors the pathways of UV irradiation giving more CO regardless of equivalent fluence. Additional studies that explore the trends presented here with different ice mixtures, photon fluxes, and fluences with a well characterized lamp are needed.

**Figure 9:**
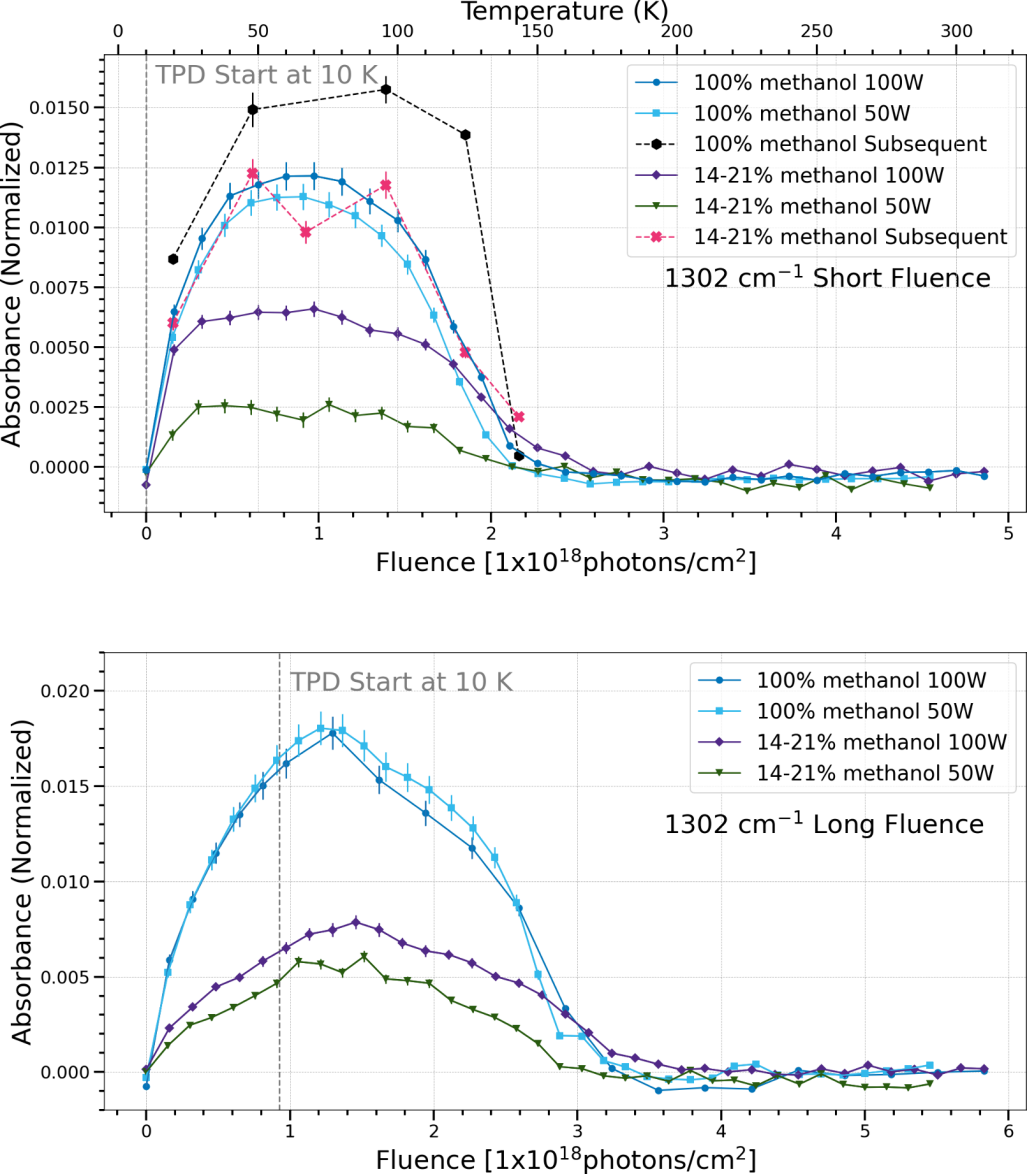
Change in normalized integrated absorbance of the 1302 cm^–1^ band as a function of UV fluence and temperature. Short fluence refers to a total UV fluence of (4.5–4.9) × 10^18^ photons cm^–2^ (top), while long fluence refers to a total UV fluence of (5.5–5.8) × 10^18^ photons cm^–2^ (bottom).

**10:**
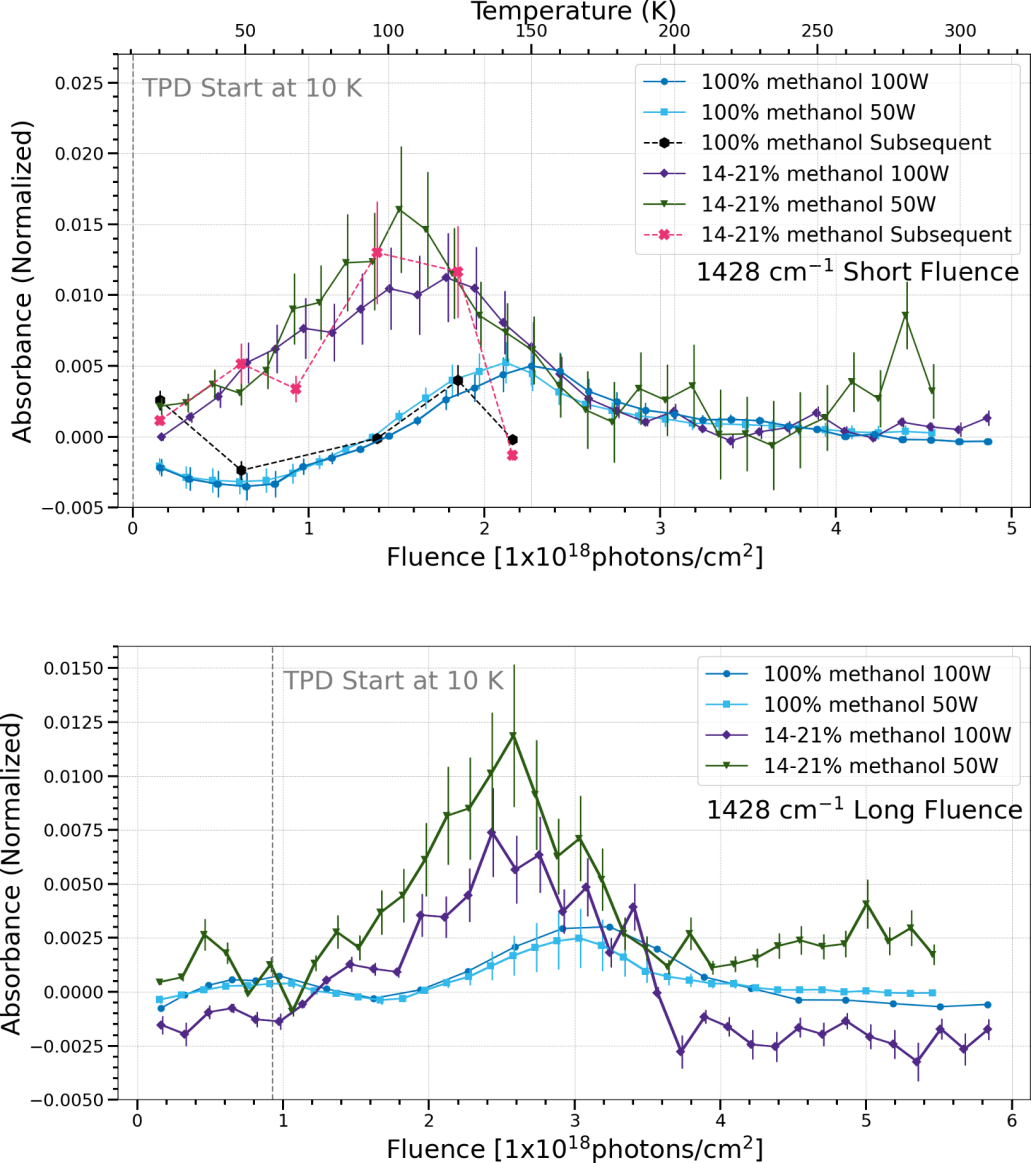
Change in normalized integrated absorbance of the 1428 cm^–1^ band as a function of UV fluence and temperature. Short fluence refers to a total UV fluence of (4.5–4.9) × 10^18^ photons cm^–2^ (top), while long fluence refers to a total UV fluence of (5.5–5.8) × 10^18^ photons cm^–2^ (bottom).

**11:**
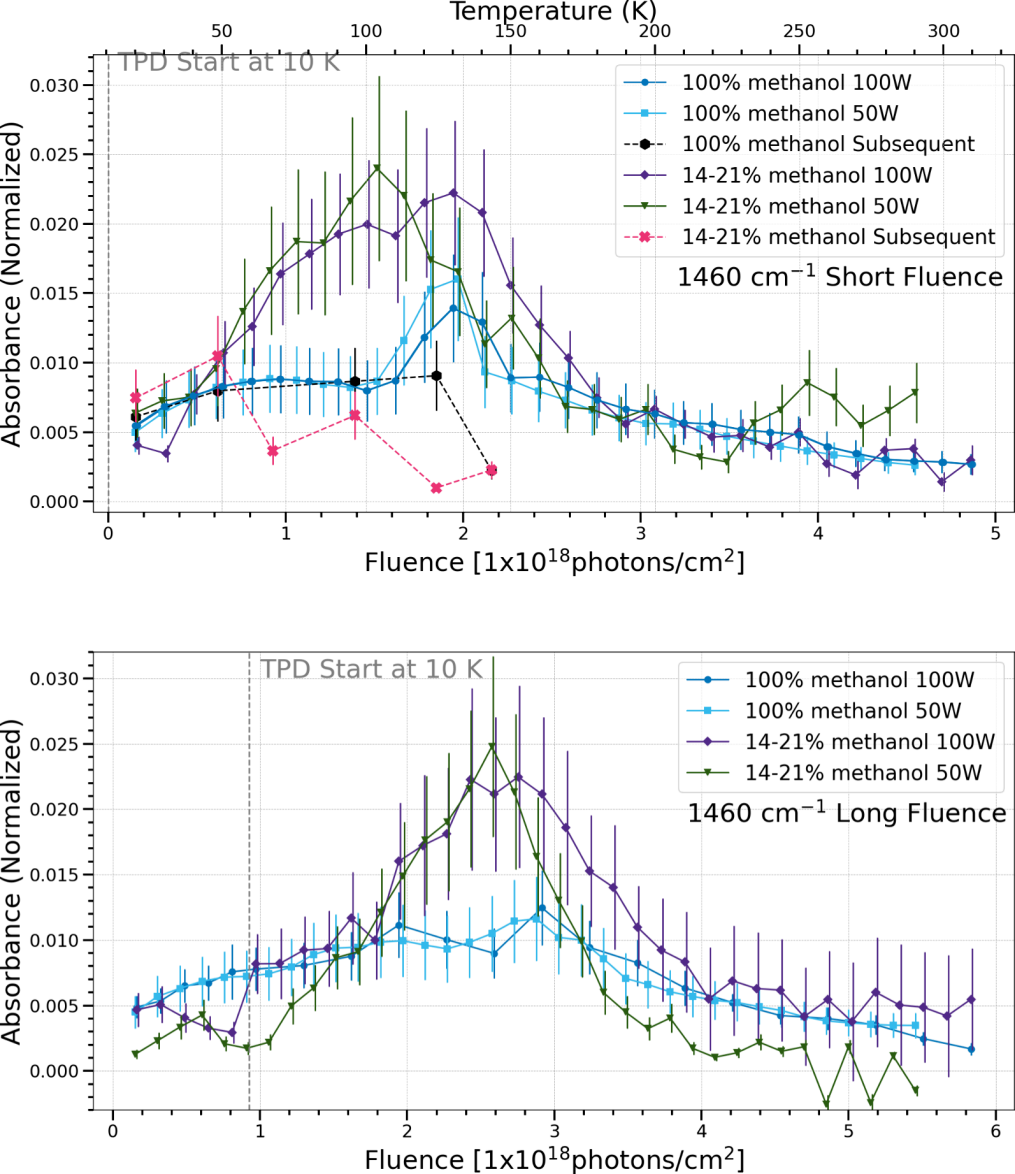
Change in normalized integrated absorbance of the 1460 cm^–1^ band as a function of UV fluence and temperature. Short fluence refers to a total UV fluence of (4.5–4.9) × 10^18^ photons cm^–2^ (top), while long fluence refers to a total UV fluence of (5.5–5.8) × 10^18^ photons cm^–2^ (bottom).

**12:**
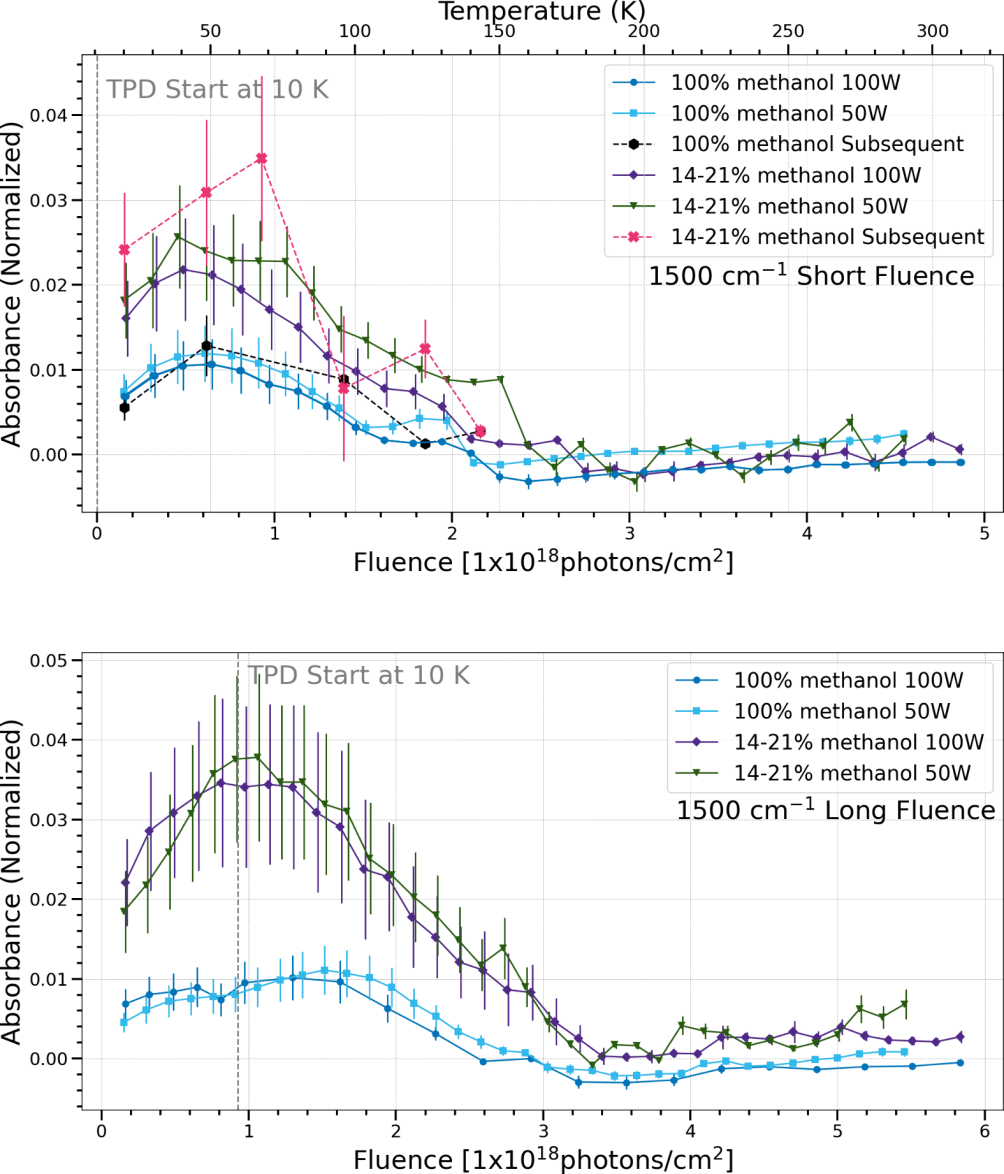
Change in normalized integrated absorbance of the 1500 cm^–1^ band as a function of UV fluence and temperature. Short fluence refers to a total UV fluence of (4.5–4.9) × 10^18^ photons cm^–2^ (top), while long fluence refers to a total UV fluence of (5.5–5.8) × 10^18^ photons cm^–2^ (bottom).

**13:**
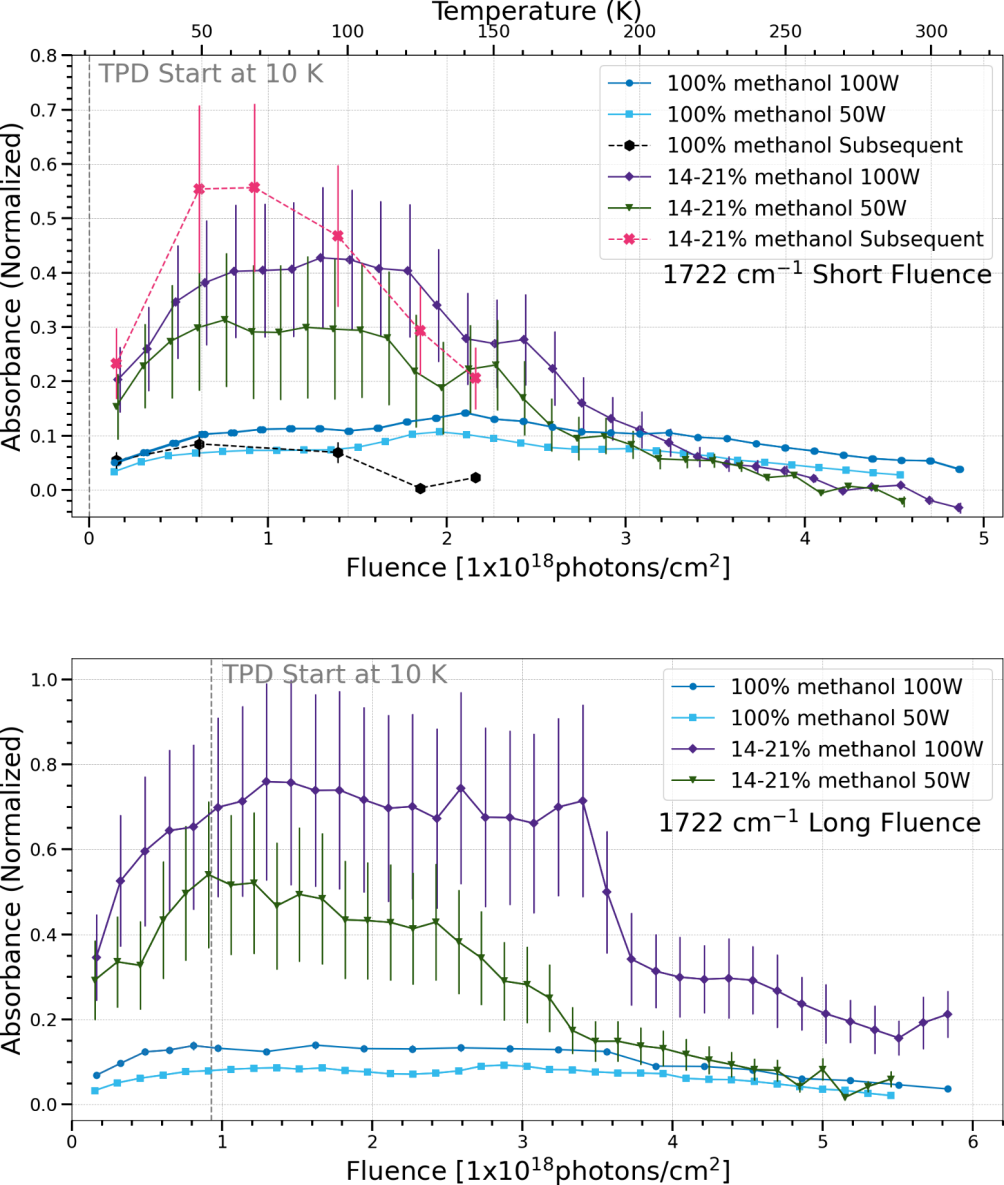
Change in normalized integrated absorbance of the 1722/1746 cm^–1^ band as a function of UV fluence and temperature. Short fluence refers to a total UV fluence of (4.5–4.9) × 10^18^ photons cm^–2^ (top), while long fluence refers to a total UV fluence of (5.5–5.8) × 10^18^ photons cm^–2^ (bottom).

**Figure 14:**
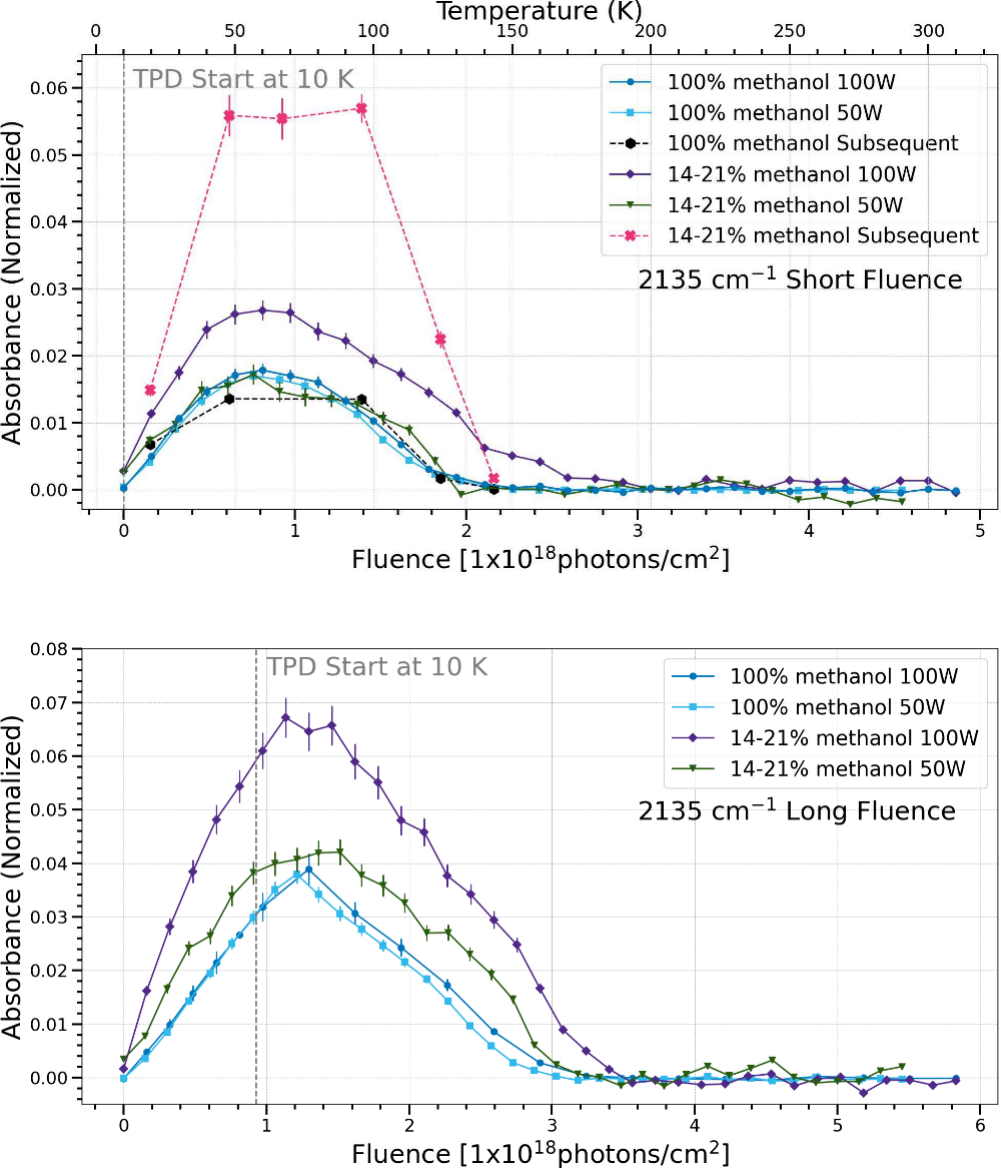
Change in normalized integrated absorbance of the 2135 cm^–1^ band as a function of UV fluence and temperature. Short fluence refers to a total UV fluence of (4.5–4.9) × 10^18^ photons cm^–2^ (top), while long fluence refers to a total UV fluence of (5.5–5.8) × 10^18^ photons cm^–2^ (bottom).

### QMS Data

During simultaneous UV irradiation and temperature-programmed desorption, quadrupole mass spectrometry data was continuously recorded to monitor the gas phase products. The QMS records mass-to-charge ratios (*m*/*z*) of all fragments from species in the gas phase from 1 to 100 amu. Mass spectrometry alone cannot be used to identify species as many of the mass fragments of interest from organics overlap. Main fragments presented here come from water (18, 17, 16 *m*/*z*) and methanol (29, 31, 15, 32, 14, 28, 13, 30, 12, 33 *m*/*z*). Here we list mass fragments in decreasing intensity according to their fragmentation patterns observed from our QMS instrument. TPD curves of the subsequent processing trials are not equivalent at all points with the simultaneous processing trials, and thus are omitted from the analysis. All QMS data weresmoothed for clarity using a moving average set to an interval of 10 data points.

Figure 15 shows the mass-to-charge ratios of gas phase products of a pure methanol ice during simultaneous irradiation and TPD. Figure 16 shows the mass-to-charge ratios of gas phase products in a 16 ± (4)% methanol in water ice. Each particular temperature on the *x*-axis corresponds to a UV fluence value as shown on the productions plots (Figures 5–14). The pure methanol ice trials were dominated by mass fragments from methanol while the methanol and water mixed ices were dominated by water fragments with some methanol fragments. All other QMS results are listed in the Supporting Information.

**Figure 15:**
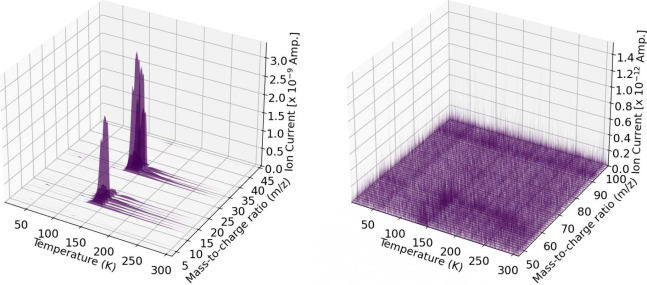
Pure methanol ice during simultaneous UV irradiation and TPD corresponding to trial 1. The *m*/*z* values are 3–46 (left) and 47–100 (right) with increasing temperature along the *x*-axis from 10 to 310 K.

**Figure 16:**
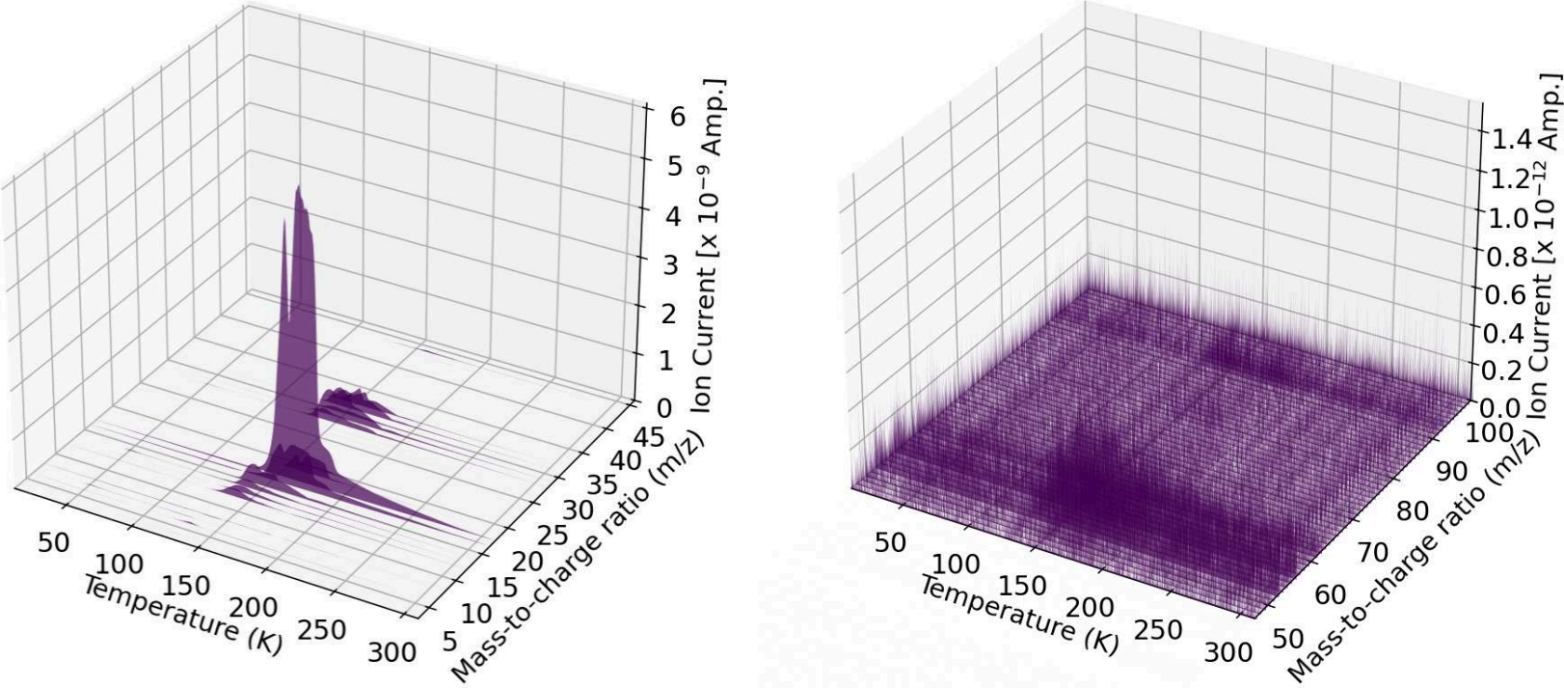
16 ± 4% methanol ice during simultaneous UV irradiation and TPD corresponding to trial 5. The *m*/*z* values are 3–46 (left) and 47–100 (right) with increasing temperature along the *x*-axis from 10 to 310 K. 18 *m*/*z* was omitted due to oversaturation of the QMS.

Figure 17 compares the temperature-programmed desorption of mass-to-charge ratio 17, which is the second highest abundance fragment of water. Using the *m*/*z* = 18 signal would be an unambiguous indication of the gas phase water present, but is not shown because the signal hit the high sensitivity limit of the QMS. However, peak desorption temperatures for each case correlate well between *m*/*z* = 17 and 18, thus allowing 17 to be used as a proxy for the water content. Other species such as formic acid could share this mass fragment if present,^44^ but this is not likely since no IR features of that species were observed. The peak desorption temperature of *m*/*z* = 17 in the pure methanol ices is approximately the same at 144–149 K with similar desorption curves for all trials. This signal is also most likely due to water since the photolysis of pure methanol produces water.^7^ In trials with methanol and water ice mixtures, the peak desorption temperature was much higher at 167–168 K, but the TPD curves were similar regardless of flux or fluence. These peak desorption temperatures for both ice compositions coincided with bulk ice sublimation.

**Figure 17:**
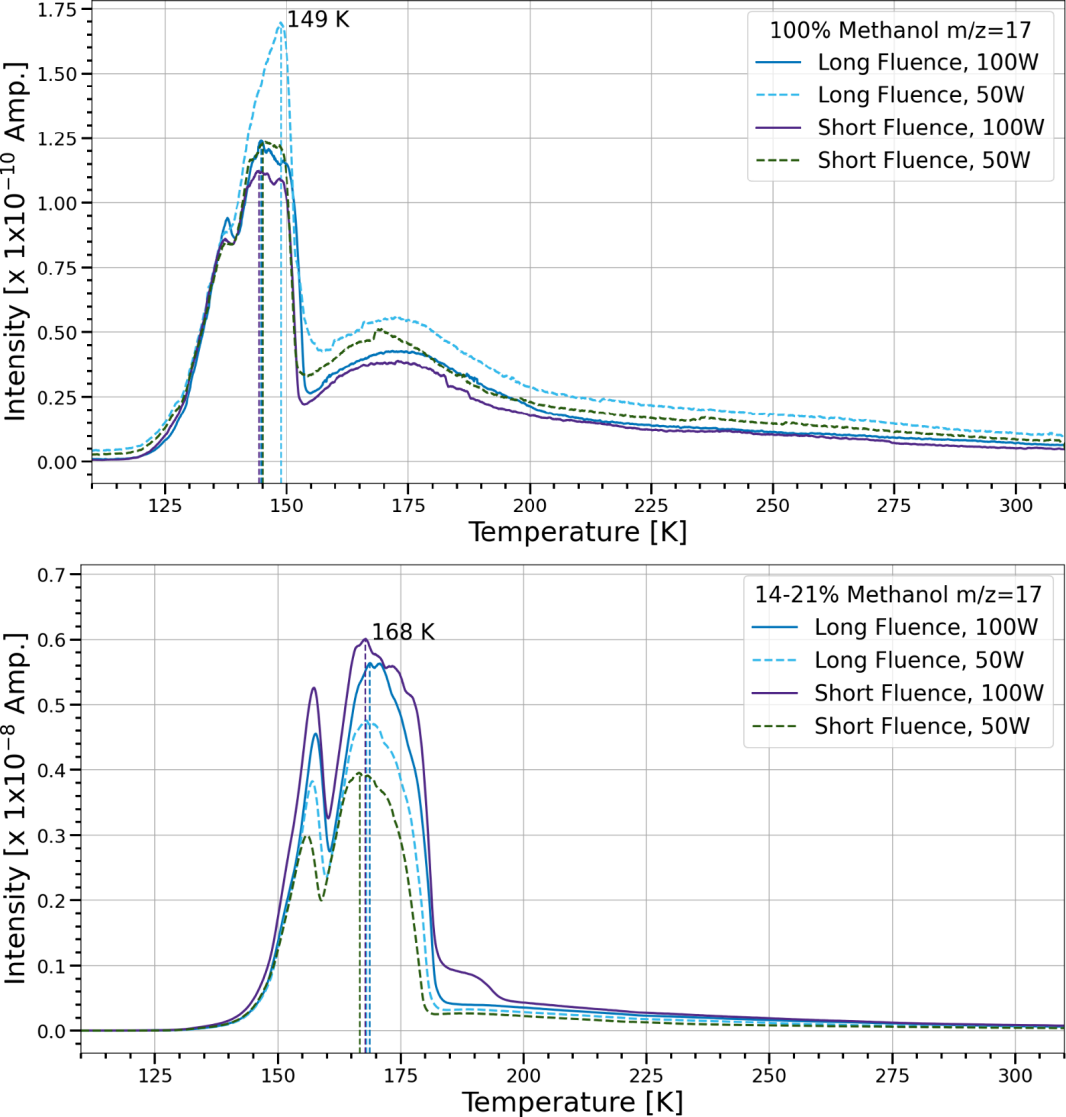
TPD of *m*/*z* = 17 for pure methanol and mixtures in the simultaneous trials. The ion current intensity (amps) of the *m*/*z* signal is plotted as a function of temperature (K). The temperature range shown is from 110 to 310 K. Dashed lines correspond to the peak desorption temperature for each trial.

Figure 18 shows the TPD curves of *m*/*z* = 29, the fragment of methanol with the largest signal. This signal shows similar trends as in Figure 17. In the pure methanol ices, the peak desorption temperatures cluster around 145 K with similar TPD curves regardless of flux or fluence. This peak desorption temperature is slightly higher than the 139 K value seen in previous work.^25^ This trace has a peak desorption temperature at 154–157 K in methanol and water ice mixtures regardless of flux or fluence. Again, this is a higher peak desorption temperature of comparable methanol and water mixtures of 10% at 146 K.^25^ The TPD curves are similar, with the exception of a double peak of almost equal intensity earlier at 143 K, due to a structural change in the ice from amorphous to crystalline. This crystallization process can also be seen as a change of band shape in the O–H and C–H vibrational mode of methanol.^45^ In any case, the signal for a *m*/*z* of 29 had a peak desorption temperature that coincided with bulk ice sublimation for both ice compositions.

**Figure 18:**
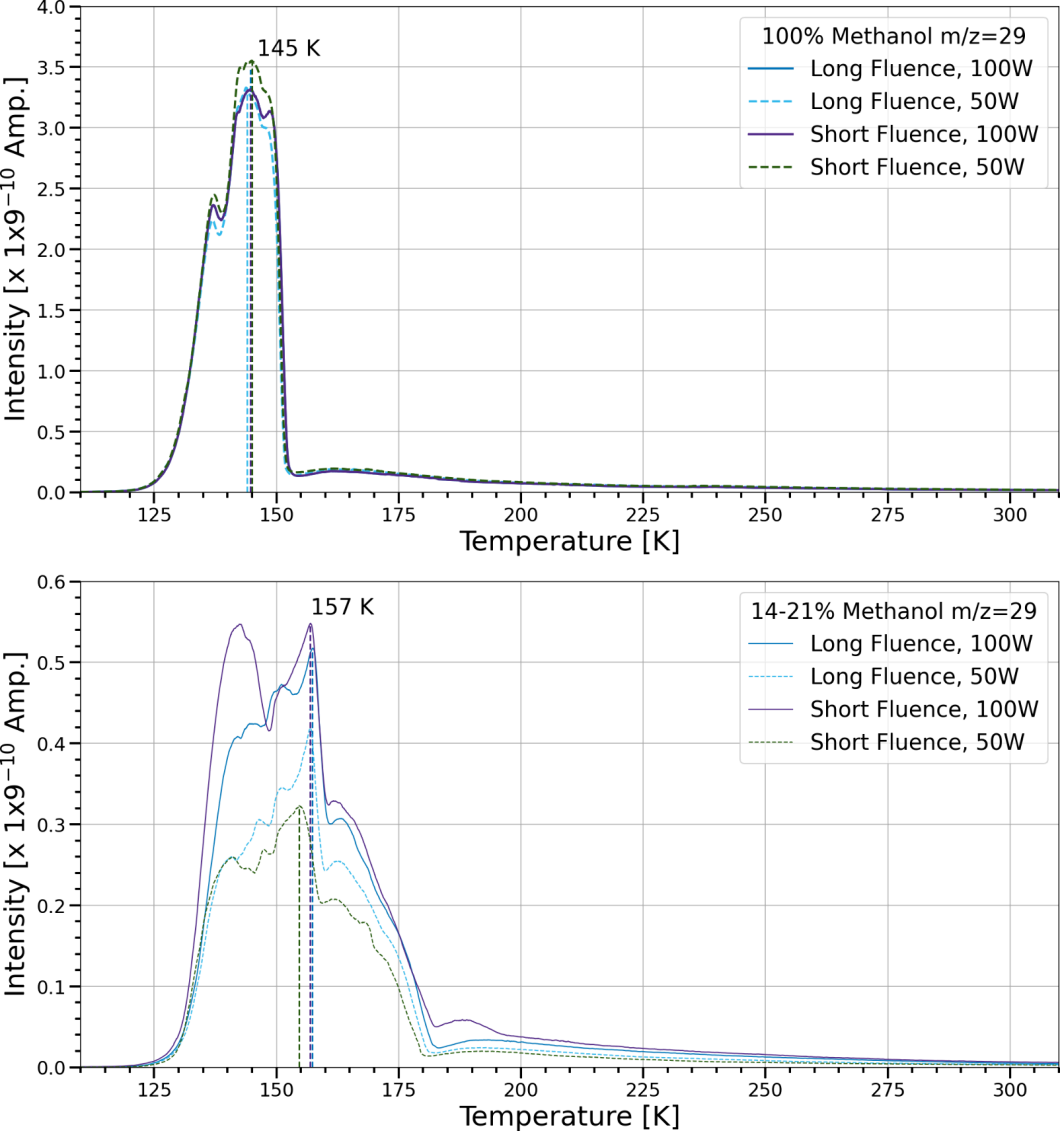
TPD of *m*/*z* = 29 for pure methanol and mixtures in the simultaneous trials. The ion current intensity (amps) of the *m*/*z* signal is plotted as a function of temperature (K). The temperature range shown is from 110 to 310 K. Dashed lines correspond to the peak desorption temperature for each trial.

Based on the major species in the QMS traces for the simultaneous processing trials, the effect of flux on the peak desorption temperatures is minimal. Regardless of flux, both ice compositions studied here resulted in approximately the same desorption temperature for methanol and water, and did not significantly impact when the bulk ice sublimation occurs. A larger factor for when the bulk ice sublimates remains the ice composition as a whole, with increasing water content leading to higher peak desorption temperatures of the major ice constituents in agreement with other works.^7,22,46^

## Conclusions

The first goal of this study was to examine the impact of simultaneous irradiation and heating of interstellar ice analogs as compared to traditional ice experiments where irradiation was followed by subsequent heating. The motivation for this aspect of the work was to more closely mimic the processing that occurs in astronomical environments where the two processes would occur at the same time. These experiments were conducted on both pure methanol samples and methanol and water mixtures. We found that subsequent UV photoprocessing and TPD resulted in enhanced production of some simpler species such as CO, H_2_CO, and CH_4_, while increasing the destruction rate of methanol. Additionally, the trials involving subsequent processing suppressed the production of more complex species such as (CH_2_OH)_2_, HOCH_2_CHO, and CH_3_OCH_3_. This indicates that simultaneous processing involving both UV irradiation and heating leads to more complex chemistry in interstellar ices for both pure methanol and mixed methanol and water ices. This effect is best explained by the enhanced mobility of larger radicals with simultaneous heating yielding more complex molecules. In trials involving subsequent irradiation and heating, the photoproducts are trapped at 10 K in binding sites before heating is applied, leading to simpler products.

It became clear during initial investigations of mixed water and methanol ices that changes in the resultant chemistry arose when there were changes in the flux of the UV lamp, even when fluence was held constant. This goes against an assumption often made in the field of laboratory astrochemistry, namely that the chemistry is reproducible if the fluence is held constant between experiments. This assumption is important for comparison between different experiments either within one laboratory or across multiple laboratories. Furthermore, this assumption is what allows laboratory experiments to be compared to astronomical observations, since the UV flux in the ISM is 10 orders of magnitude less than typical laboratory conditions. Results from this study show that in experiments involving methanol and water mixed ices, the resultant chemistry may change when comparing experiments with different lamp fluxes, regardless of whether the fluence is held constant. This is particularly true for methanol, CO, and CH_4_ in the case of water and methanol ice mixtures. These observations are most likely explained by the favoring of particular formation pathways. We did confirm that the chemistry remains unchanged between experiments with the same fluence for all species in a pure methanol ice and for a subset of other species in mixed methanol and water ices. Additional studies with a well characterized MDHL UV lamp are needed to explore this trend with other ice mixtures, photon flux, and fluences.

## Supplementary Material



## Notes

Experimental ice compositions and thicknesses, IR spectra for the simultaneous trials, additional IR normalized production plots, and additional QMS plots of experimental trials (PDF)
