# Supplementary material for: Laboratory Investigation of Simultaneous Ultraviolet Photoprocessing and Temperature-Programmed Desorption of Interstellar Ice Analogs
Source: ACS Earth Space Chem. 2026 Jan 30;10(2):536–54. doi: 10.1021/acsearthspacechem.5c00338 (PMC12927026; doi:10.1021/acsearthspacechem.5c00338)
Supplement: Supplementary file 1 [file sp5c00338_si_001.pdf]

Laboratory Investigation of Simultaneous Ultraviolet Pholeftrocessing and Temperature-Programmed Desorption of Interstellar Ice Analogs

Collette C. Sarver,† Catherine E. Walker,† and Susanna L. Widicus Weaver*,‡

†*Department of Chemistry, University of Wisconsin-Madison, Madison, WI, USA*

‡*Departments of Chemistry and Astronomy, University of Wisconsin-Madison, Madison, WI, USA*

E-mail: slww@chem.wisc.edu

Supporting Information for Publication

Table S1: Experimental ice samples used in the analysis. Trial type refers to the experimental conditions found in Table 1 and 2. 1.62×10^{-17} cm/molecule was used as the band strength for the C-O vibrational mode for the pure methanol ices[1]. That band strength was reduced to 1.42×10^{-17} cm/molecule in the 14-21% methanol and water ices[1]. 1.2×10^{-17} cm/molecule was used as the band strength for the bending vibrational mode of water at 1660 cm^{-1}[2].

**Trial type**	**Methanol Content (%)**	**Water Content (%)**	**Total Column Density (molecules cm^{-2})**
1	100	0	$9.2(\pm0.3) \times 10^{17}$
2	100	0	$9.2(\pm0.3) \times 10^{17}$
2	100	0	$7.6(\pm0.3) \times 10^{17}$
2	100	0	$8.2(\pm0.3) \times 10^{17}$
3	100	0	$9.8(\pm0.3) \times 10^{17}$
3	100	0	$9.1(\pm0.3) \times 10^{17}$
3	100	0	$9.6(\pm0.3) \times 10^{17}$
3	100	0	$9.5(\pm0.3) \times 10^{17}$
4	100	0	$9.2(\pm0.3) \times 10^{17}$
4	100	0	$8.7(\pm0.3) \times 10^{17}$
5	16(±4)	84(±4)	$9(\pm2) \times 10^{17}$
5	19(±4)	81(±4)	$9(\pm2) \times 10^{17}$
6	16(±4)	84(±4)	$1.1(\pm0.3) \times 10^{18}$
6	14(±3)	86(±3)	$9(\pm2) \times 10^{17}$
6	15(±4)	85(±4)	$1.0(\pm0.2) \times 10^{18}$
6	14(±3)	86(±3)	$1.0(\pm0.2) \times 10^{18}$
6	14(±3)	85(±3)	$1.1(\pm0.3) \times 10^{18}$
7	15(±4)	85(±4)	$7(\pm2) \times 10^{17}$
7	15(±4)	85(±4)	$1.0(\pm0.2) \times 10^{17}$
8	14(±3)	86(±3)	$1.0(\pm0.2) \times 10^{17}$
9	100	0	$7.3(\pm0.2) \times 10^{17}$
10	100	0	$7.9(\pm0.3) \times 10^{17}$
11	100	0	$9.6(\pm0.3) \times 10^{17}$
12	100	0	$9.5(\pm0.3) \times 10^{17}$
13	100	0	$8.5(\pm0.3) \times 10^{17}$
14	17(±6)	83(±6)	$1.0(\pm0.2) \times 10^{18}$
15	18(±4)	82(±4)	$8(\pm2) \times 10^{17}$
16	15(±3)	85(±3)	$9(\pm2) \times 10^{17}$
17	15(±3)	85(±3)	$9(\pm2) \times 10^{17}$
18	21(±5)	79(±5)	$1.0(\pm0.2) \times 10^{18}$
19	20(±4)	80(±4)	$9(\pm2) \times 10^{17}$

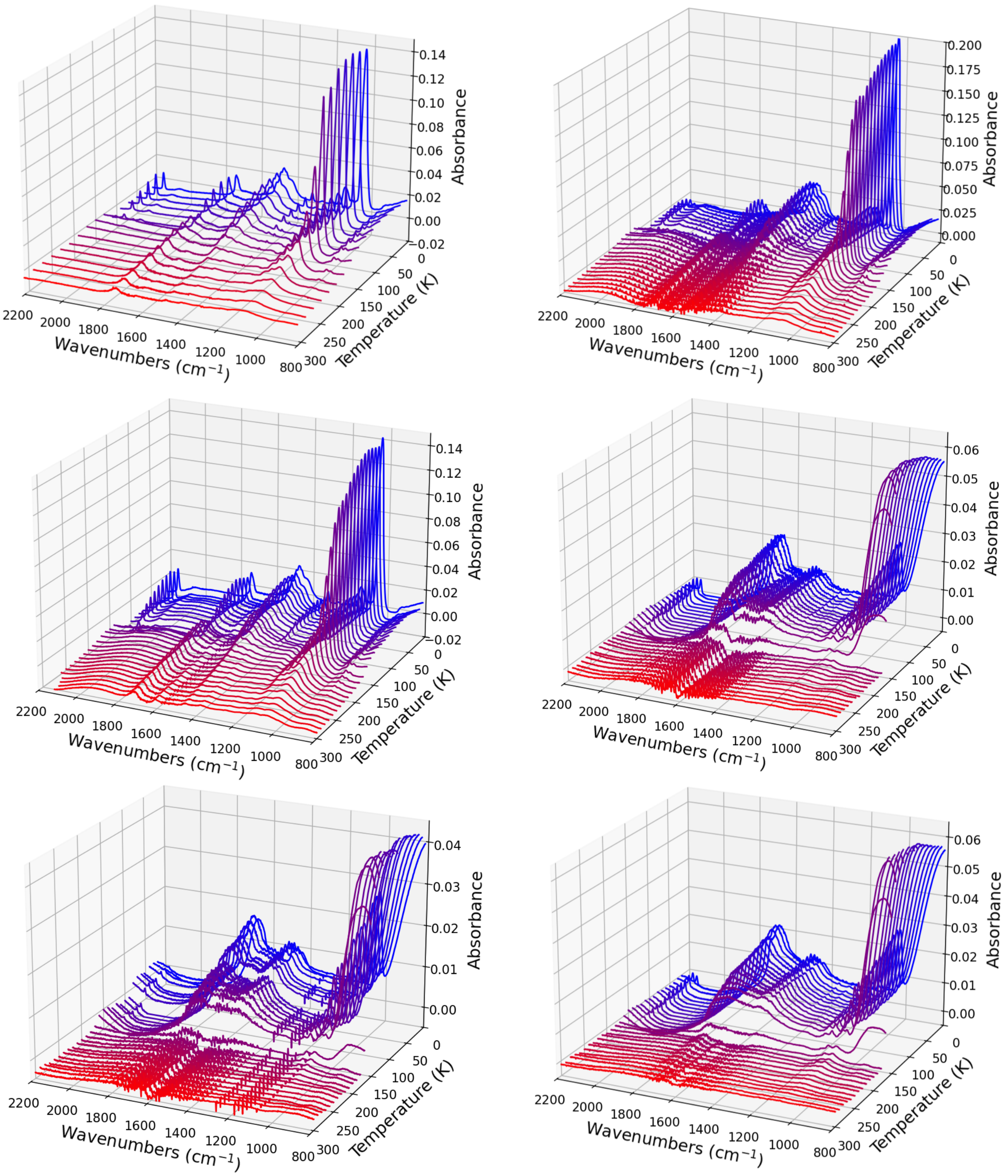

Figure S1: Infrared spectra of pure methanol ices (first 3) and 14-21% methanol in water mixed ices (last 3) from 10 K to 310 K under simultaneous UV pholeftrocessing and TPD, corresponding to trials 2-4 and 5-8. The wavelength range is from 800 to 2200 cm^{-1} for clarity.

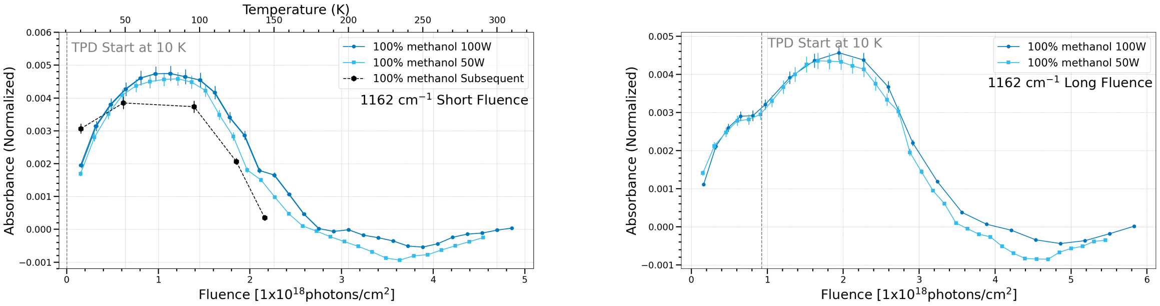

Figure S2: Change in normalized integrated absorbance of the 1162 cm^{-1} band as a function of UV fluence and temperature. This band was only present in the pure methanol ices. Short fluence refers to a total UV fluence of $4.5 - 4.9 \times 10^{18}$ photons cm^{-2} (left) while long fluence refers to a total UV fluence of $5.5 - 5.8 \times 10^{18}$ photons cm^{-2} (right).

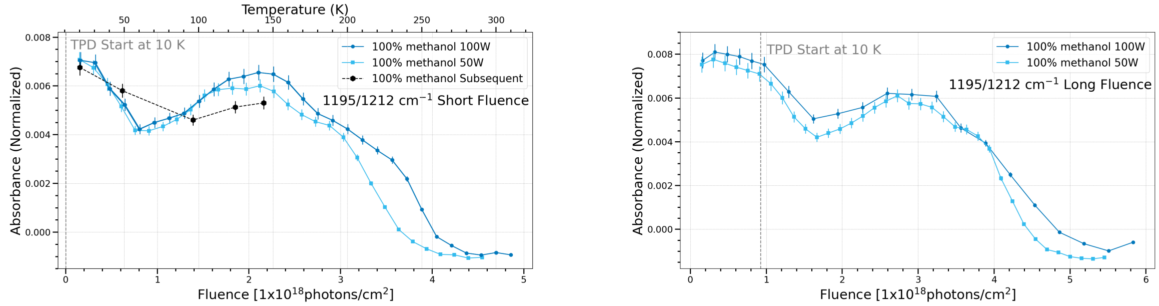

Figure S3: Change in normalized integrated absorbance of the 1195/1212 cm^{-1} bands as a function of UV fluence and temperature. These bands were only present in the pure methanol ices. Short fluence refers to a total UV fluence of $4.5 - 4.9 \times 10^{18}$ photons cm^{-2} (left) while long fluence refers to a total UV fluence of $5.5 - 5.8 \times 10^{18}$ photons cm^{-2} (right).

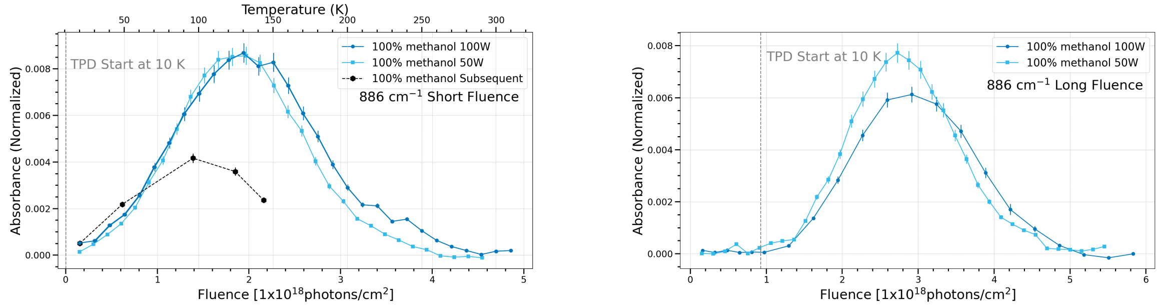

Figure S4: Change in normalized integrated absorbance of the 886 cm^{-1} band as a function of UV fluence and temperature. These bands were only present in the pure methanol ices. Short fluence refers to a total UV fluence of $4.5 - 4.9 \times 10^{18}$ photons cm^{-2} (left) while long fluence refers to a total UV fluence of $5.5 - 5.8 \times 10^{18}$ photons cm^{-2} (right).

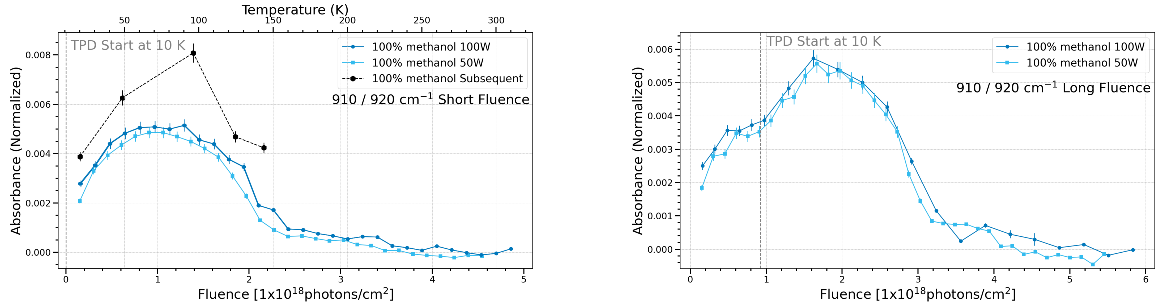

Figure S5: Change in normalized integrated absorbance of the 910/920 cm^{-1} bands as a function of UV fluence and temperature. These bands were only present in the pure methanol ices. Short fluence refers to a total UV fluence of $4.5 - 4.9 \times 10^{18}$ photons cm^{-2} (left) while long fluence refers to a total UV fluence of $5.5 - 5.8 \times 10^{18}$ photons cm^{-2} (right).

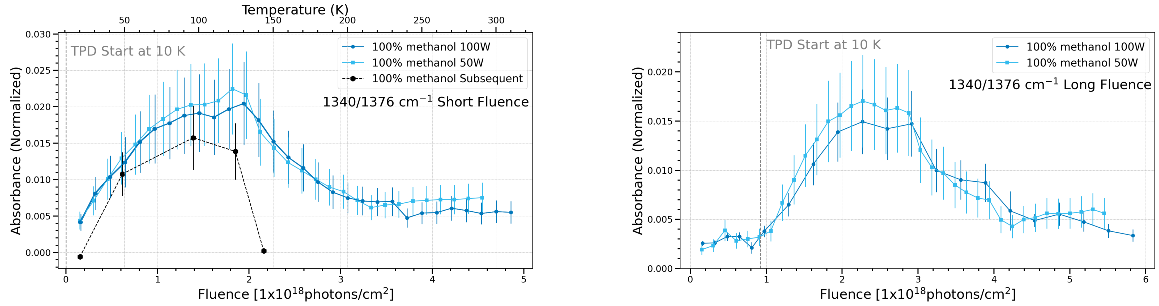

Figure S6: Change in normalized integrated absorbance of the 1340/1376 cm^{-1} bands as a function of UV fluence and temperature. These bands were only present in the pure methanol ices. Short fluence refers to a total UV fluence of $4.5 - 4.9 \times 10^{18}$ photons cm^{-2} (left) while long fluence refers to a total UV fluence of $5.5 - 5.8 \times 10^{18}$ photons cm^{-2} (right).

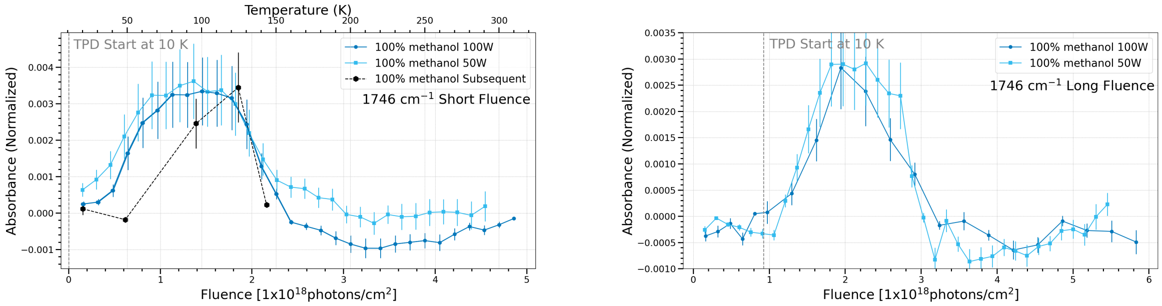

Figure S7: Change in normalized integrated absorbance of the 1746 cm^{-1} band as a function of UV fluence and temperature. This band was only present in the pure methanol ices. Short fluence refers to a total UV fluence of $4.5 - 4.9 \times 10^{18}$ photons cm^{-2} (left) while long fluence refers to a total UV fluence of $5.5 - 5.8 \times 10^{18}$ photons cm^{-2} (right).

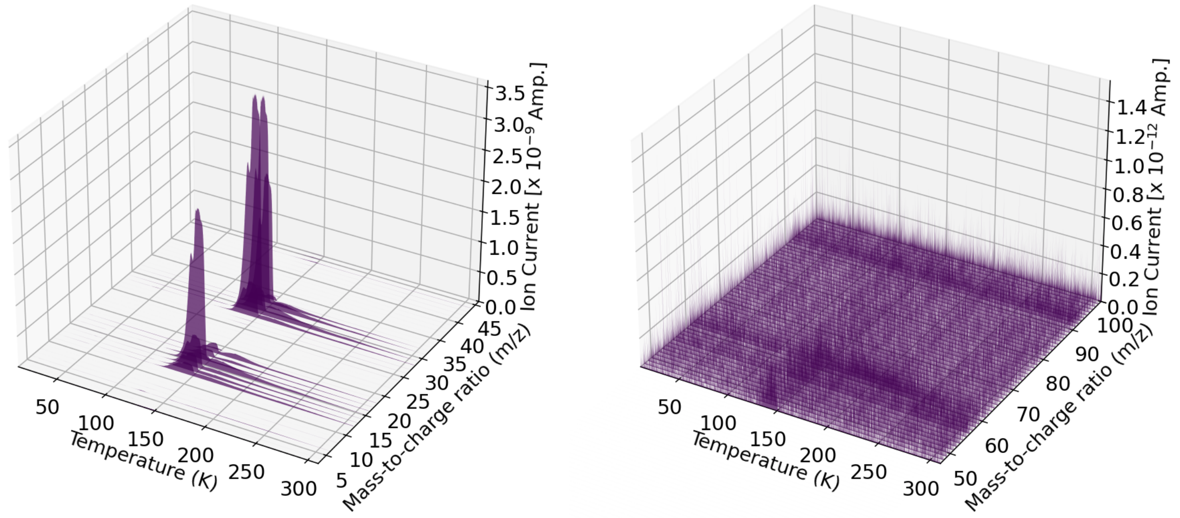

Figure S8: Pure methanol ice during simultaneous UV irradiation and TPD corresponding to trial 2. The m/z values are 3-46 (left) and 47-100 (right) with increasing temperature along the x-axis from 10 to 310 K.

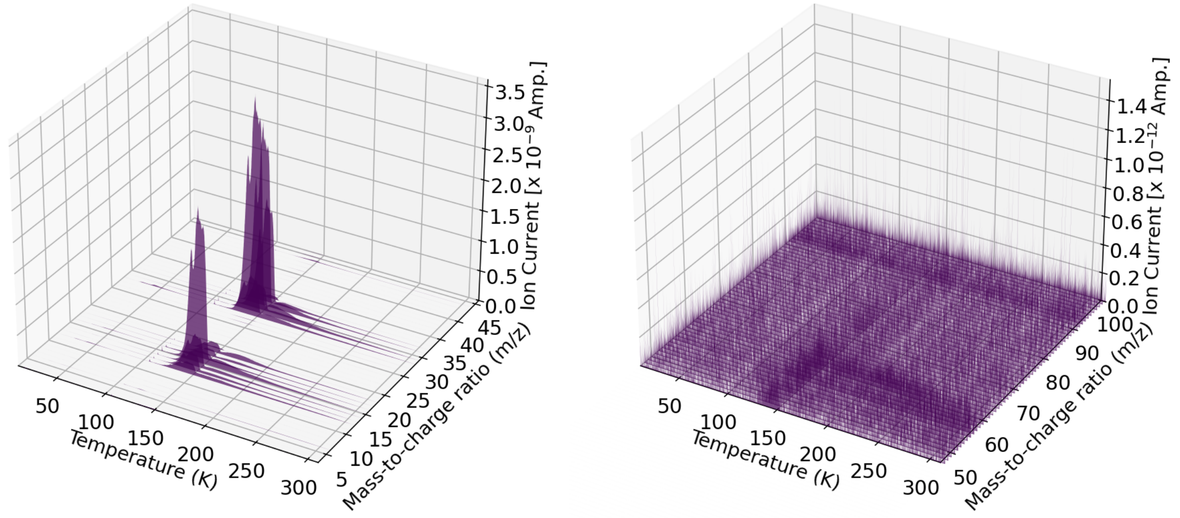

Figure S9: Pure methanol ice during simultaneous UV irradiation and TPD corresponding to trial 3. The m/z values are 3-46 (left) and 47-100 (right) with increasing temperature along the x-axis from 10 to 310 K.

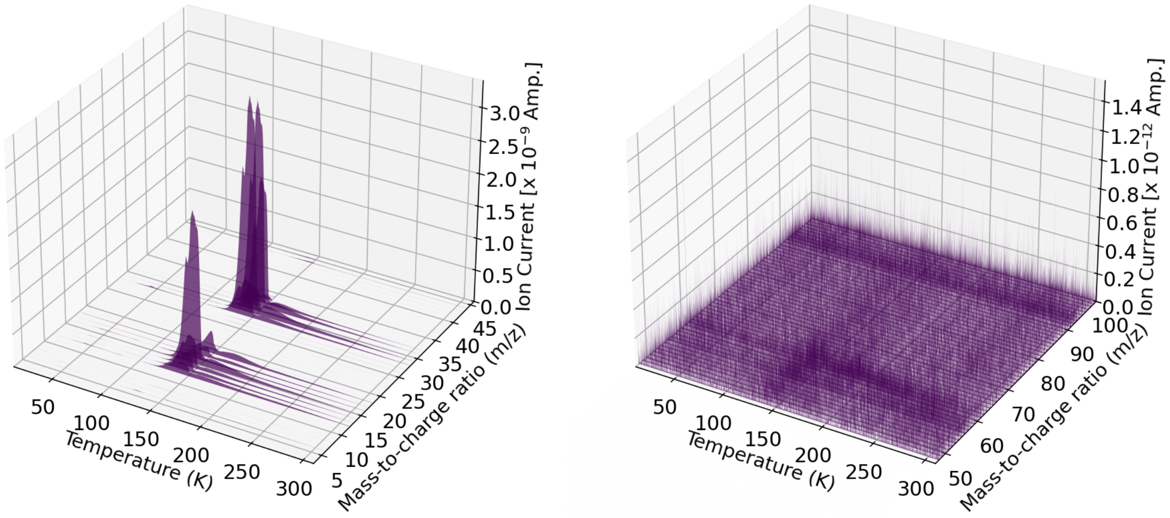

Figure S10: Pure methanol ice during simultaneous UV irradiation and TPD corresponding to trial 4. The m/z values are 3-46 (left) and 47-100 (right) with increasing temperature along the x-axis from 10 to 310 K.

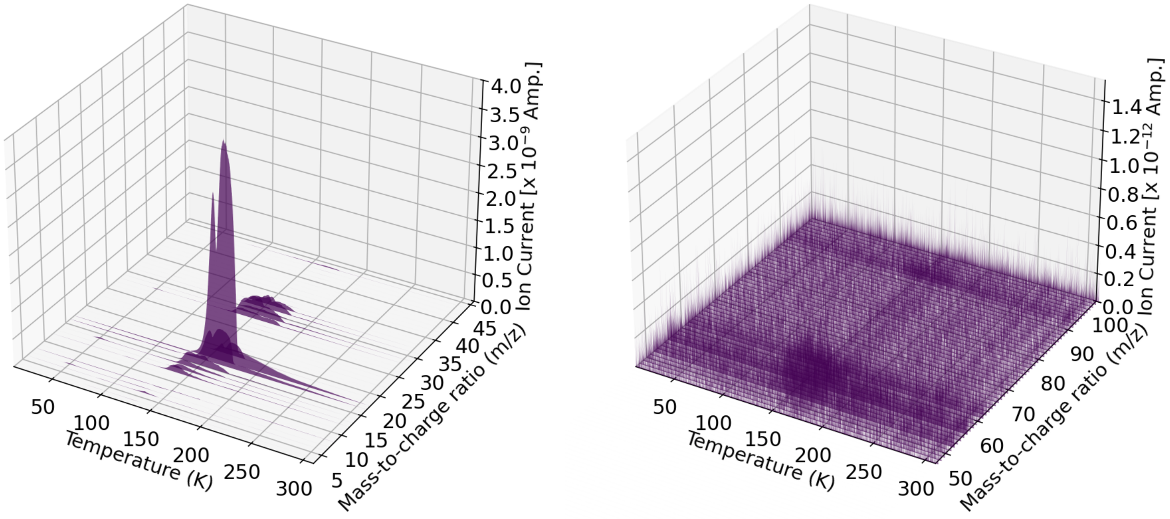

Figure S11: 15±4% methanol ice during simultaneous UV irradiation and TPD corresponding to trial 6. The m/z values are 3-46 (left) and 47-100 (right) with increasing temperature along the x-axis from 10 to 310 K. 18 m/z was omitted due to oversaturation of the QMS.

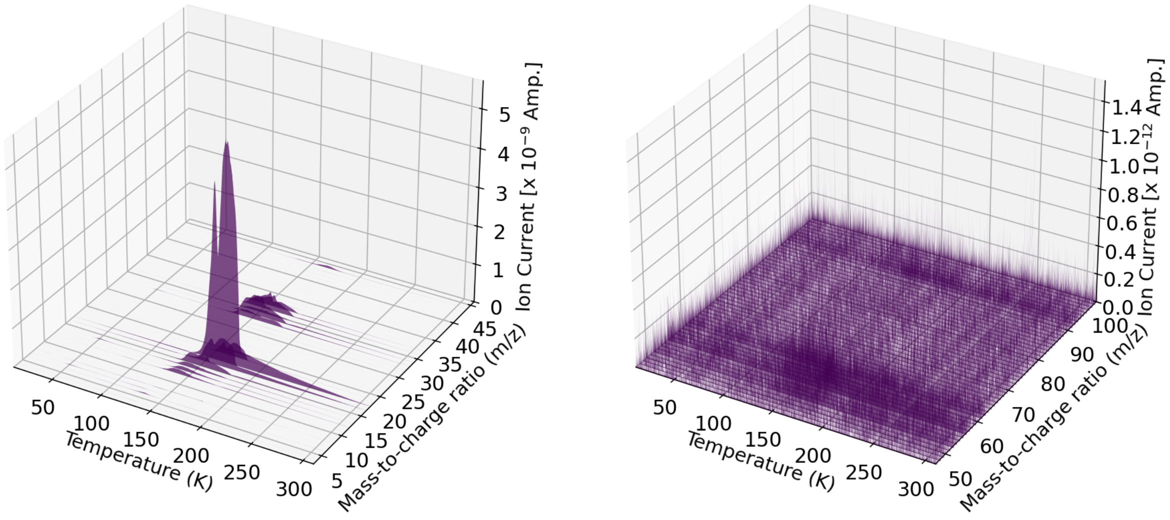

Figure S12: 15±4% methanol ice during simultaneous UV irradiation and TPD corresponding to trial 7. The m/z values are 3-46 (left) and 47-100 (right) with increasing temperature along the x-axis from 10 to 310 K. 18 m/z was omitted due to oversaturation of the QMS.

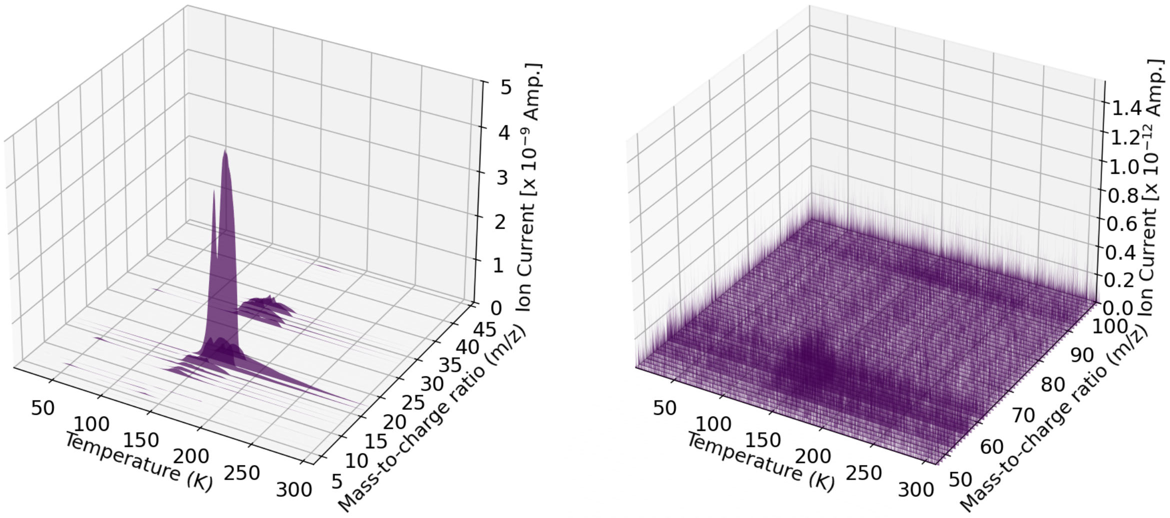

Figure S13: 14±3% methanol ice during simultaneous UV irradiation and TPD corresponding to trial 8. The m/z values are 3-46 (left) and 47-100 (right) with increasing temperature along the x-axis from 10 to 310 K. 18 m/z was omitted due to oversaturation of the QMS.

References

(1) Hudson, R. L.; Gerakines, P. A.; Yarnall, Y. Y. Infrared Spectroscopic and Physical Properties of Methanol Ices—Reconciling the Conflicting Published Band Strengths of an Important Interstellar Solid. *Astrophys. J.* **2024**, *970*, 108.

(2) Gerakines, P. A.; Schutte, W. A.; Greenberg, J. M.; van Dishoeck, E. F. The Infrared Band Strengths of H_2O, CO and CO_2 in Laboratory Simulations of Astrophysical Ice Mixtures. *Astron. Astrophys.* **1995**, *296*, 810.
